# Population Status, Ecology and Conservation Challenges of Cattle Egrets (*Bubulcus ibis*), Especially in the Southern Africa Subregion: A Systematic Review and Evidence Synthesis

**DOI:** 10.1002/ece3.74300

**Published:** 2026-09-15

**Authors:** Bhekinkosi Sibanda, Tawanda Tarakini, Josphine Mundava

**Affiliations:** ^1^ School of Wildlife, Ecology and Conservation Chinhoyi University of Technology Chinhoyi Zimbabwe; ^2^ Research Platform, Production and Conservation in Partnership Harare Zimbabwe; ^3^ Department of Forest Resources and Wildlife Management National University of Science and Technology Bulawayo Zimbabwe

**Keywords:** ecosystem services, environmental monitoring, foraging dynamics, land‐use change, One Health

## Abstract

The Cattle Egret ( *Bubulcus ibis* ) is one of Africa's most adaptable yet environmentally sensitive waterbirds, occupying natural wetlands, grazing systems, irrigated agro‐mosaics and urban green spaces. We conducted a systematic review of 173 studies published between the 1940s and 2025, incorporating global research where it provided insights relevant to Southern African populations. Using narrative synthesis and quantitative summaries, we evaluated population trends, habitat use, breeding ecology, threats and ecosystem services. Across all studies, habitat and foraging ecology dominated the literature (46.2%), followed by population status and distribution (21.4%), breeding ecology and behaviour (16.8%) and threats and contaminants (15.6%). In Southern Africa, atlas interpretations and site‐based surveys from South Africa, Zimbabwe, Botswana, Namibia and Lesotho indicate generally stable or increasing populations over the past three decades, with SABAP‐derived occupancy rising from 0.38 (1987–1991) to 0.60 (2007–present). Densities generally peak in irrigated pastures and peri‐urban green spaces of eastern South Africa, but remain low in arid Namibia, high‐altitude Lesotho and Zambia's under‐sampled floodplains. Foraging studies show diets dominated by Orthoptera, enhanced success near livestock and machinery and seasonal shifts in both diet and foraging locations that reflect hydrology‐driven changes in prey availability and habitat accessibility. Breeding studies report opportunistic, rainfall‐synchronised nesting, mean colony sizes of ~65 nests, clutch sizes of 2.9 eggs and fledging success of ~62%. Key threats include loss of nesting habitat, extreme‐weather colony failures, organochlorine contamination and pathogen spillover at poultry interfaces, while ecosystem‐service benefits include pest suppression and nutrient redistribution. We recommend three priorities for future research: (1) advanced telemetry and stable‐isotope analyses to map movements; (2) routine contaminant and pathogen screening within One Health frameworks; and (3) expanded atlas‐based and citizen‐science monitoring to address geographic bias. These steps will clarify the species' dual role as a service provider and early‐warning indicator of environmental change across Southern Africa.

## Graphical abstract

The Cattle Egret ( *Bubulcus ibis* ) occupies a wide range of natural and human‐modified habitats, yet research effort across its range is highly uneven. Our systematic review of 173 studies synthesises patterns in population trends, habitat use, breeding ecology, threats and ecosystem services, revealing strong geographic and thematic biases in available evidence. We highlight key conservation gaps and recommend targeted monitoring, contaminant screening and movement ecology studies to strengthen regional management across Southern Africa.

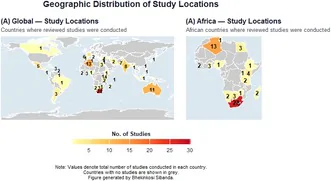

## 1 Introduction

Human‐driven land‐use change is rapidly reshaping ecosystems across Southern Africa. Expanding urban centres, intensified agriculture, transport corridors and extractive industries continue to convert natural habitats and fragment once‐continuous landscapes (Ogutu et al. 2011; Parnell et al. 1988). This fragmentation disrupts nutrient flows, alters ecological interactions and erodes biodiversity (Burkepile et al. 2013), producing complex mosaics in which remnant natural patches are interwoven with farmland, settlements and modified wetlands. Such patchy landscapes tend to favour adaptable generalist species while disadvantaging habitat specialists which are dependent on large, connected ecosystems. Recent regional syntheses show that land‐use intensification alters avian functional diversity and ecosystem processes across African rangelands and agro‐ecosystems (Sirami et al. 2019). Yet, despite numerous site‐specific investigations, no comprehensive synthesis has evaluated how contrasting land‐use regimes shape Cattle Egret ( *Bubulcus ibis* ) populations across Southern Africa, leaving a critical gap in understanding how landscape transformation influences this widely distributed species.

Among the avifauna navigating shifting environments, the Cattle Egret provides a compelling model for examining species' responses to environmental change. The species comprises of three subspecies: *B. i. ibis* (Africa, W. Asia, S. Europe, the Americas); *B. i. coromandus* (E. Asia to Australasia/Oceania); and *B. i. seychellarum* (Seychelle)—though taxonomic boundaries remain debated (BirdLife International 2024; Clements et al. 2023; del Hoyo 2008; Gill et al. 2024; Hoyo et al. 1992). The Cattle Egret is originally native to Africa (Cramp et al. 1988; Maddock and Geering 1994), and it has expanded to every continent except Antarctica within just over a century, from its first extra‐African colonisations in the late 19th century to its current global distribution (Miño et al. 2022). Although extensively studied in West and East Africa, Europe, Asia and the Americas; research effort within Southern Africa remains comparatively limited. This global‐regional mismatch underscores the need for a focused synthesis that identifies local ecological patterns, knowledge gaps and conservation priorities that may be obscured in broader global reviews.

Ecologically, the Cattle Egret functions as a versatile meso‐level consumer, foraging opportunistically on invertebrates, particularly those flushed by large mammals and potentially reducing ectoparasite loads on its hosts (Dinsmore 1973; Grubb 1976). In agricultural landscapes, these behaviours can deliver pest‐suppression services, while in natural rangelands they may reflect herbivore abundance and movement patterns, making the species a potential bioindicator of rangeland health (Savory and Butterfield 2016). However, its indicator value is context‐dependent: although the Cattle Egret distribution often tracks herbivore presence, sensitivity to contaminants, hydrological alteration and disturbance suggests it can also reflect broader environmental change (Bouwman et al. 2019; Caron et al. 2014). Increasing reliance on anthropogenic food sources in peri‐urban contexts, such as landfill sites, may elevate exposure to pathogens and intensify human–wildlife health interfaces (BirdLife International 2014b; Cele et al. 2025; Craig et al. 2021). Declines in large wild herbivores across parts of East and Southern Africa (Ogutu et al. 2014) further raise concerns about the long‐term stability of these commensal relationships.

Although current population trends for Cattle Egrets in Southern Africa are generally stable or increasing (Bowker and Downs 2012; Cele et al. 2025), historical and regional variability persists. Some areas experienced declines during the 1980s and early 1990s, and recent increases appear strongest in eastern South Africa and parts of Zimbabwe, while other regions show weak or inconsistent trajectories (Underhill et al. 2016). The species' ecological breadth (Kushlan 2012; Telfair 1981), close association with human‐modified landscapes (Louw 2005; Tarboton et al. 1987), and provision of key ecosystem services (Andiatsirevombola et al. 2016; Green and Elmberg 2013) make it an important focal species for understanding biodiversity responses to rapid environmental change. As a widespread, well‐monitored generalist (Maddock and Geering 1994; Underhill et al. 2016), it offers a sensitive lens on emerging pressures including contaminants (Bouwman, Kylin, et al. 2013; Bouwman, Viljoen, et al. 2013; Bouwman et al. 2019), extreme weather (Bowker and Downs 2012; McKilligan 1987), and disease (McKilligan 1987; Møller et al. 2009) that may also affect less adaptable waterbirds. Its continued expansion into agricultural and urban systems (Cele et al. 2025; Craig et al. 2021; Louw 2005) creates opportunities through pest suppression and nutrient cycling (Maddock 1989; Markwell and Driessen 1990; Siegfried 1971b) and risks through increased exposure to pollutants and disturbance (BirdLife International 2014b; Bouwman, Kylin, et al. 2013; Bouwman, Viljoen, et al. 2013). Studying the Cattle Egret therefore provides a model for how adaptable species navigate socio‐ecological transformation, yielding insights relevant to integrated land‐use planning, wetland management and One Health surveillance (Caron et al. 2014; Savory and Butterfield 2016).

Despite extensive site‐based research on the species' distribution, foraging, and breeding ecology, no region‐wide synthesis has critically examined how contrasting land‐use regimes shape Cattle Egret populations across Southern Africa. This absence constrains conservation managers' ability to monitor population trends, identify principal threats, and integrate the species' ecological role into land‐use and biodiversity planning. The challenge is particularly acute in poorly studied areas, notably Zambia, Angola, Namibia and Mozambique where ecological baselines are less documented despite extensive wetland systems. Practitioners in these regions often rely on broad regional or continental patterns that often obscure local variation, limiting the precision of conservation assessments and management decisions.

In this review, we consolidate current knowledge on population status, ecology, land‐use associations and conservation challenges; map research coverage to show spatial and thematic gaps; evaluate the quality, relevance and consensus of existing studies; and highlight practical, high‐impact entry points for targeted research, monitoring and conservation action. By integrating ecological, threats and habitat‐specific perspectives, the synthesis provides an evidence base to guide biodiversity management, sustainable agriculture and One Health surveillance. Structured around four analytical pillars: (i) population status and distribution, (ii) habitat use and foraging ecology, (iii) breeding ecology and behaviour and (iv) conservation challenges, ecosystem services and management implication, the review positions the Cattle Egret as both a model for understanding avian resilience and vulnerability in rapidly changing landscapes and as a sentinel and service provider whose conservation is directly relevant to ecosystem integrity and human livelihoods.

Throughout this review, ‘Southern Africa’ follows the SADC regional definition, encompassing Angola, Botswana, Comoros, the Democratic Republic of Congo, Eswatini, Lesotho, Madagascar, Malawi, Mauritius, Mozambique, Namibia, Seychelles, South Africa, Tanzania, Zambia and Zimbabwe. Although centred on Southern Africa, the synthesis incorporates global studies where they provide ecological, behavioural, movement or conservation insights relevant to regional populations. By structuring the review around four analytical pillars, we contextualise regional patterns within the species' global ecology while maintaining a clear focus on Southern Africa landscapes.

## 2 Materials and Methods

We conducted a systematic review following the ROSES (Reporting Standards for Systematic Evidence Syntheses) protocol and checklist (Haddaway et al. 2018), ensuring transparent documentation of study identification, screening eligibility assessment and inclusion. The PRISMA 2020 (Preferred Reporting Items for Systematic Reviews and Meta Analyses) (Page et al. 2021) was employed to structure the reporting flow within the ROSES framework. We recorded all ROSES required metadata to support transparency and reproducibility (Haddaway et al. 2018). We developed the review in accordance with the Collaboration for Environmental Evidence (CEE) Guidelines (CEE 2013). Although our primary geographic focus was Southern Africa, we also included studies from outside the region when they provided ecological, behavioural, movement, or conservation insights directly relevant to the Cattle Egret in Southern Africa or offered useful comparative context.

### 2.1 Analytical Framework

We organised the synthesis around four predefined pillars:

- *Population status and distribution*: trends in abundance, occupancy and range shifts from local to regional scales
- *Habitat use and foraging ecology*: habitat associations, seasonal movements, prey composition and foraging strategies.
- *Breeding ecology and behaviour*: nesting substrates, colony structure, timing, productivity and drivers of breeding success.
- *Conservation challenges, ecosystem services and management implications*: documented pressures (e.g., habitat loss, contaminants, disease, extreme weather), quantified ecosystem functions (e.g., pest suppression, nutrient cycling) and their relevance for management and policy.

To align with ROSES guidance (Haddaway et al. 2018), we structured the review question using standard evidence‐synthesis components:

- Population: Cattle Egrets ( *Bubulcus ibis* ) globally, with a primary focus on Southern Africa.
- Exposure/Context: habitat, land‐use, climate, ecological and anthropogenic conditions.
- Comparator: Temporal or spatial contrasts where applicable.
- Outcomes: population trends, habitat associations, breeding metrics, threats and ecosystem functions.

The pillars provided guided data extraction coding and narrative synthesis, enabling us to identify cross‐cutting ecological patterns, knowledge gaps and evidence clusters.

### 2.2 Search Strategy

We conducted the systematic review searches between July 2025 and March 2026, combining the scientific and common names of the Cattle Egret ( *B. ibis* ) with terms relating to population status, ecology, habitat and land‐use associations, breeding, threats and conservation in Southern Africa. We adapted Boolean operators and wildcards to each database. We applied an English‐language filter in the search in both Scopus and Web of Science.

We used the Scopus and Web of Science bibliographic databases as they are the two large multidisciplinary sources and provide comprehensive global coverage of peer‐reviewed ecological literature. Although these platforms index a wide range of document types including research articles, book chapters, conference proceedings, letters, meeting abstracts, notes, short communications and occasional non‐scientific formats, we included only those records that met the predefined eligibility criteria as outlined in the section below. The breadth increases search sensitivity and reduces the risk of missing relevant ecological studies, consistent with ROSES recommendations for comprehensive and transparent searching (Haddaway et al. 2018).

We also applied ROSES‐aligned supplementary search techniques, including backward and forward citation tracking, screening bibliographies of relevant books and theses, and searching institutional repositories and grey‐literature sources. These steps helped capture regionally relevant ecological studies not indexed in major databases, consistent with best practice in environmental evidence synthesis (Haddaway et al. 2018). We did not manually search Google Scholar because of its non‐transparent ranking algorithm, lack of reproducibility, and inconsistent metadata conflict with ROSES and PRISMA methodological standards (Haddaway et al. 2018; Page et al. 2021). We imported both sets of results exactly as retrieved, with no modification to date coverage.

The full database‐specific search strings are provided in Section S1, and the complete list of included studies is provided in Section S3.

### 2.3 Eligibility Criteria

Studies were included if they reported primary or secondary ecological data on Cattle Egret ( *Bubulcus ibis* ) within any Southern African states (Angola, Botswana, Comoros, the Democratic Republic of Congo, Eswatini, Lesotho, Madagascar, Malawi, Mauritius, Mozambique, Namibia, Seychelles, South Africa, Tanzania, Zambia and Zimbabwe) and addressed at least one analytical pillar. We also included university theses, books, and grey‐literature reports when they presented original ecological data with clear methods and extractable results.

We included studies from outside Southern Africa when they provided ecological, behavioural, migratory, or conservation insights directly relevant to Southern African Cattle Egret populations. We excluded experimental and quasi‐experimental studies, captive or ex situ studies, anecdotal records without supporting evidence, and studies lacking primary ecological data or sufficient methodological detail.

### 2.4 Screening and Selection

We imported all records from Scopus (exported as a .bib file) and Web of Science (exported as plain text) into a reference manager and then into the R language for statistical computing (R Core Team 2024) for screening. We conducted screening and coding in R to ensure reproducibility, version control, and transparent documentation of decisions. We identified 589 records (Web of Science: 257; Scopus: 332 post‐filter). After merging datasets using the *bibliometrix* R package, we removed 31 duplicates, leaving 558 unique records.

We screened titles first as an umbrella triage step, as this provided a clear and efficient way to eliminate obviously irrelevant studies before progressing to abstract and full‐text screening. We excluded 190 records at this stage, while 146 were coded ‘Yes’ and 222 ‘Maybe’, resulting in 368 records progressing to abstract screening. Of these, 40 lacked abstracts, 161 were coded ‘Yes’, 161 ‘No’ and 6 ‘Maybe’. We excluded the 161 ‘No’ records based on geographic, taxonomic or thematic mismatch. We took the remaining 207 records (40 blanks, 161 Yes, 6 Maybe) forward for full‐text assessment.

**FIGURE 1:**
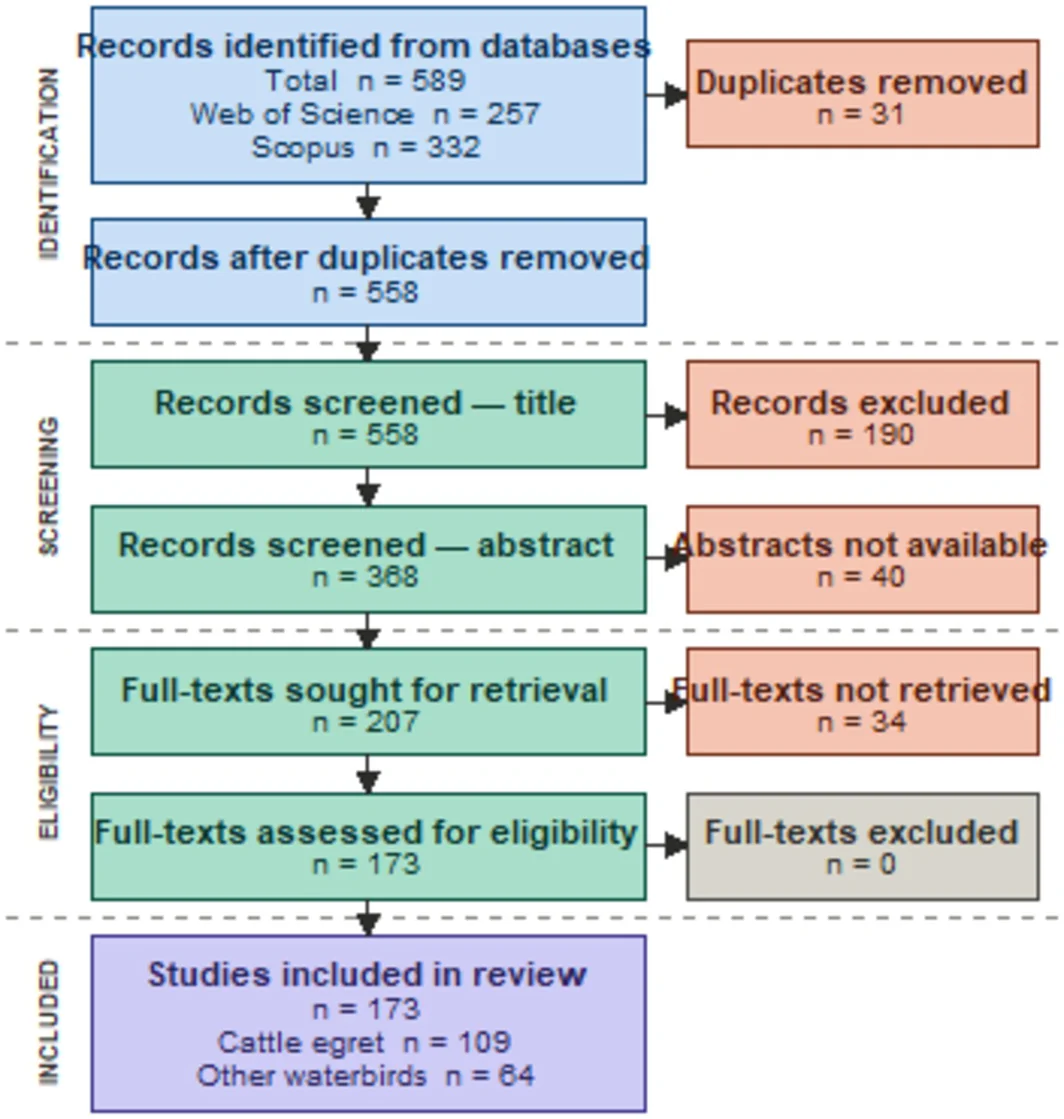
PRISMA (2020) flow diagram summarising the identification, screening, eligibility assessment and inclusion of studies in the systematic review.

We sought full texts for all 207 records; however, 34 could not be retrieved despite multiple institutional access routes and repeated search attempts and were therefore excluded. We screened the remaining 173 full texts in detail, and all met the eligibility criteria. Of these, 109 studies focused exclusively on cattle egrets or included them as a primary study species, forming the core evidence base. The remaining studies examined broader waterbird assemblages but contained relevant Supporting Information on cattle egrets. The full selection process is summarised in Figure 1 (PRISMA flow diagram) and detailed in Section S2.

### 2.5 Data Extraction and Coding

We extracted bibliographic details, study location (country and coordinates where available), habitat or land‐use context, study period and sampling effort, methodological approach and key findings relevant to the four analytical pillars. We did not work with raw datasets from the Southern African Bird Atlas Project (SABAP1 or SABAP2). Instead, we extracted only the interpreted outputs reported in peer‐reviewed publications and atlas summaries. We coded each study to one or more analytical pillars based on the ecological information reported, enabling thematic synthesis and identification of cross‐cutting patterns. Study level metadata (Country, institutions, year, study region and study type) are summarised in Table S4 and all R scripts used for screening, deduplication, PRISMA generation and mapping are provided in Supporting Information S5.

### 2.6 Evidence Assessment

We classified each study's contribution according to the nature of the evidence (supporting, mixed or none), its strength (strong or weak) and its type (direct or circumstantial). We documented patterns of concordance and conflict to gauge consistency across studies and identify where evidence converged or diverged.

### 2.7 Risk of Bias

We evaluated potential sources of bias, including variation in sampling design, effort, and timing; geographic imbalance in study coverage (with a concentration of studies in South Africa and Zimbabwe and under‐representation of Angola, Namibia, and Mozambique); and methodological variability in survey techniques, analytical approaches, and reporting standards. We also accounted for uneven availability of grey literature across countries, a recognised challenge in environmental evidence synthesis and one reason ROSES‐aligned supplementary searches were incorporated. These factors were used to weigh confidence in thematic patterns and to highlight priority knowledge gaps for future research.

## 3 Results

This systematic review incorporated 173 studies that met the eligibility criteria and provided extractable ecological information on the Cattle Egret. Out of these, 109 studies focused directly on Cattle Egrets, while 64 studies examined broader waterbird assemblages but contained relevant supplementary data. The evidence base spans more than seven decades of research, from early colony establishment records in the 1940s and 1950s (Siegfried 1965, 1968; Skead 1952) and extending to contemporary studies published between the early 2000s and 2025, including atlas‐based assessment (Taylor et al. 2019; Underhill et al. 2016), urban ecology analyses (Cele et al. 2025; Craig et al. 2021), and research on agricultural landscape dynamics (Padhiary et al. 2025; Sirami et al. 2019).

Geographically, the studies included were unevenly distributed across Southern Africa (Figure 3). Research effort was concentrated in South Africa (75%) and Botswana (8.3%), with additional contributions from Namibia (5.6%), Madagascar (5.6%), Zimbabwe (2.8%) and Lesotho (2.8). No peer‐reviewed studies were conducted in Mozambique; however, several South African studies included observations or ecological notes from Mozambican localities, meaning that Mozambique was represented indirectly rather than through dedicated research. Notably, no peer‐reviewed primary ecological data were available for major wetland systems in Zambia, including the Bangweulu Wetlands, Kafue Flats, and Barotse Floodplain, creating a clear geographic evidence gap.

**FIGURE 2:**
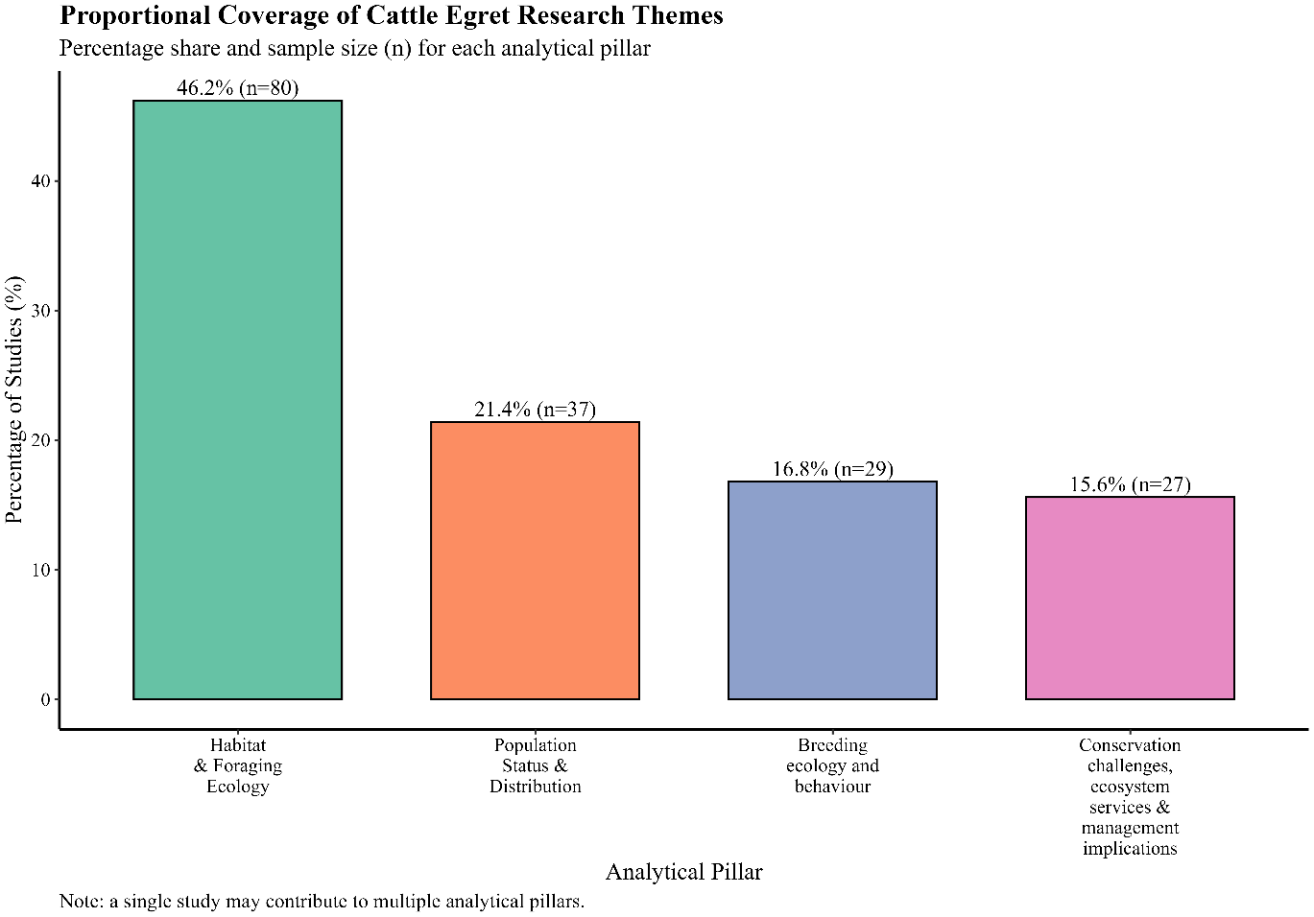
Proportional coverage of Cattle Egret research themes across four analytical pillars. The chart displays the percentage share of all analytical pillar tags.

Thematically, studies contributed to one or more of the four predefined analytical pillars. Habitat use and foraging ecology dominated the literature (46.2%), followed by population status and distribution (21.4%), breeding ecology and behaviour (16.8%), and studies focused on threats, contaminants or ecosystem‐services dynamics (15.6%) as shown in Figure 2 below. Although many studies contributed to multiple pillars, each study was assigned to the pillar representing its dominant thematic emphasis to avoid double‐counting in proportional summaries.

Collectively, the included studies provide a diverse and regionally rich evidence base, enabling synthesis of long‐term population trends, habitat associations, breeding ecology, and emerging conservation challenges for the species across Southern Africa.

### 3.1 Population Status and Distribution

The Cattle Egret exhibits a dynamic, complex global population status and distribution, characterised by rapid range expansion, opportunistic colonisation, and significant population fluctuations. While its native range largely encompasses Africa, the species has successfully colonised new areas, including the Americas and Australia, demonstrating remarkable adaptability to diverse environments (Beckett 1965).

The global expansion of the Cattle Egret is driven by its exceptional capacity for rapid colonisation and ecological adaptability (Bowker and Downs 2012). In Southern Africa, the species shifted from being rare to established within a five to ten years during the 1940s and early 1950s, with numbers increasing rapidly as suitable foraging habitats expanded and mixed‐species heronries provided social cues that facilitated settlement. Early records show the species to be uncommon in the King William's Town area in the Eastern Cape Province of South Africa before 1941, nest numbers had risen sharply from 429 in 1949 to 669 in 1950, with the total colony exceeding 2000 birds (Siegfried 1965, 1971b; Skead 1952, 1966), This illustrates both the speed and scale of establishment. Similar trajectories have been recorded globally (Arendt 1988; Maddock and Geering 1994). In Guyana, Cattle Egrets came to dominate long‐established mixed heronries within two decades of arrival, comprising 1597 of 1786 nest (≈90%) in the Botanic Gardens colony by 1959 (Lowe‐McConnell 1967), while in North America the species expanded steadily, reaching Canada by 1975 (Putnam 1978) and penetrating arid northern Mexico by the early 1970s (Zimmerman 1973). These patterns reflect an ‘internal range expansion’ similar to leap‐frog urbanisation observed in other species (Evans et al. 2010).

Social cues from established ardeid colonies frequently facilitate colonisation. In Arkansas, for example, a heronry initiated by Little Blue Herons ( *Egretta caerulea* ) expanded rapidly after Cattle Egrets joined, increasing from 1200 to 2800 individuals between 1983 and 1985 (Belzer and Lombardi 1989). Such interspecific social facilitation likely contributes to successful establishment in Southern Africa, where mixed‐species colonies are common (Bowker and Downs 2012; Kopij and Paxton 2018, 2019).

Southern African studies reveal consistent evidence of population growth and stable colony formation. Several thousand birds have been recorded in the South‐western (Siegfried 1971b; Kopij 2017), and communal roosts typically contain 1000–2000 individuals, occasionally reaching 5000 (Siegfried 1971a; Kopij 2017). These increases occurred independently of tick abundance, indicating that population growth was driven by broader ecological factors rather than ecto‐parasitism. Evidence from Australia supports this interpretation: although ticks caused nestling mortality, they did not regulate colony‐level population growth (McKilligan 1987). These structured roost networks demonstrate extensive landscape use and the species' ability to exploit heterogeneous environments (Cele et al. 2025; Craig et al. 2021). Key population metrics from regional studies are summarised in Table 1.

**TABLE 1:** Extracted population metrics from regional studies reporting colony sizes, roosting aggregations, and distributional patterns of Cattle Egrets in Southern Africa and neighbouring regions.

Study	Location	Key population finding (Southern Africa)	Quantitative data
Skead (1952, 1966)	King William's Town, South‐eastern Cape Province, South Africa	Significant population increase and colony establishment.	429 nests (1949), 669 nests (1950); estimated 2000+ birds at peak.
Siegfried (1972a, 1972b)	Paarl, South‐western Cape Province, South Africa	Large breeding colony.	Colony of approximately 8000 Cattle Egrets.
Bowker and Downs (2012)	KwaZulu‐Natal, South Africa	Widespread breeding activity, often in mixed colonies.	9 breeding events across 7 sites (1920–2010); 41 of 47 egret breeding records in mixed‐species colonies.
Siegfried (1968, 1971a)	South‐western Cape Province, South Africa	Large non‐breeding roosts.	Roosts typically 1000–2000 birds; max 5000; roosts > 10,000 are rare.
Caron et al. (2014)	Zimbabwe	Dominant species in shared habitats.	Cattle Egrets, Red‐billed Queleas, and Barn Swallows represented 65% of co‐occurrences between waterfowl and domestic bird sites.
Fasina et al. (2014)	Nigeria (West Africa)	Widespread distribution in Sub‐Saharan Africa, linked to transhumance.	Group of approximately 1000 Cattle Egrets was observed.

Across Southern Africa, published atlas interpretations and colony‐level studies provided further evidence of sustained population growth. Interpreted outputs from SABAP1 and SABAP2 analyses reported an increase in the reporting rate (i.e., the probability of recording the species per pentad) of Cattle Egret presence from 0.38 (±0.09 SD) in the 1980s to 0.60 (±0.07 SD) by the 2010s, representing a 58% relative gain (Underhill et al. 2016). KwaZulu‐Natal pentads showed the steepest increase, from 0.35 (±0.08) to 0.65 (±0.06), with peri‐urban sites around Pietermaritzburg and Durban Bay driving this trend (Kopij 2014; Kopij and Paxton 2018). Nest counts in Durban municipal parks also increased by 28% over 15 years, from a mean colony size of 12.4 ± 2.6 pairs in 1995 to 15.9 ± 3.5 pairs in 2010 (Bowker and Downs 2012).

In Mpumalanga's irrigated pastures, reporting rates rose from 0.29 to 0.55 between atlas phases (Kopij 2014), while urban parks in Gauteng increased from 0.40 to 0.75 (Kopij and Paxton 2018). Zimbabwe's Chiredzi River floodplain showed a 30% rise in colony size, from 28.5 ± 6.2 pairs in 1980 to 36.9 ± 8.1 pairs in 1990 (Tarboton et al. 1987). In Mozambique's Chókwè irrigation scheme, atlas‐style assessment by Kopij (2014) indicated that Cattle Egret occupancy doubled from 0.22 in 1991 to 0.45 by 2016. Namibia's central plateau recorded a 39% increase in roost counts (180 in 1980 vs. 250 in 2000), with this pattern supported by earlier work (Siegfried 1965, 1971a; Kopij and Paxton 2018), although occupancy remained low (< 0.18) along the western Etosha Pan margins (Kopij and Paxton 2019). Botswana's Moremi Game Reserve grew from 130 to 380 breeding pairs between 1971 and 1999 (Kopij and Paxton 2018), while high‐altitude Lesotho maintained small, fluctuating colonies (10 ± 4 pairs annually), constrained by harsh winters (Kopij 1999).

Expansion into anthropogenic habitats, including sugarcane estates on the Umfolozi flats, peri‐urban parks along the Vaal and Crocodile rivers, and dairy pastures in the Eastern Cape further illustrated the species' ecological plasticity (Cele et al. 2025; Louw 2005). However, neither peer‐reviewed studies nor the additional grey literature sources provided primary or secondary data for Zambia's major Ramsar wetlands, including the Bangweulu Wetlands, Kafue Flats, and Barotse Floodplain, leaving the country's population status undocumented and biassing regional syntheses towards better‐studied neighbours (BirdLife International 2014a; Taylor et al. 2019).

These patterns indicate that Cattle Egrets thrive in high‐productivity, human‐modified landscapes but remain constrained in arid and under‐sampled regions. Complementary evidence from outside Southern Africa report colonial peaks exceeding 300 pairs on predator‐free islands in Lake Nasser (Hering, Fischer, Geiter, et al. 2020; Hering, Fischer, Fuchs, et al. 2020) and ring‐recovery distances of up to 3750 km from South Africa into central and East Africa, while coastal populations remain largely sedentary (e.g., Kopij 2017). These spatial patterns, closely link to habitat associations and foraging strategies, provided a natural transition to the next thematic pillar.

Compared with the generally stable and increasing populations of Cattle Egret in Southern Africa (Bowker and Downs 2012; Cele et al. 2025; Craig et al. 2021), population trends elsewhere in the world are more variable. In Jamaica Bay, New York, Cattle Egrets declined significantly between 1974 and 1997 (Brown et al. 2001), and the wider Long Island population fell from 350 pairs in 1985 to 42 pairs by 1995. These declines highlight the potential for local contractions within an overall expanding global range.

Conversely, newly colonised regions frequently exhibit rapid growth. A monospecific colony in Algeria expanded to ~750 pairs by 2014 (Metallaoui et al. 2020), while mixed colonies in Iran contained substantial numbers of Cattle Egret nests (Ashoori and Barati 2013). Seasonal fluctuations are pronounced in migratory populations. In India, numbers ranged from 392–1539 individuals in 2004–2005 and 260–960 in 2005–2006, peaking from January to April and disappearing during the June–September breeding season (Seedikkoya and Azeez 2009).

Population estimates for colonial waterbirds, including Cattle Egrets, vary substantially depending on the survey method employed (Parkes et al. 2012; Prosser et al. 2022). A comparative study in Maryland demonstrated that Unmanned Aerial System (UAS drone) photo counts and flush counts underestimated White Egret pairs by 62%–68%, while flight‐line surveys overestimated pairs by 12%–57% relative to ground‐based nest counts (Prosser et al. 2022). In this study, White Egrets referred to Snowy Egrets ( *Egretta thula* ) and Cattle Egrets ( *B. ibis* ) combined, because the two species could not be distinguished in aerial imagery. This means that although the estimates were not species‐specific, the methodological patterns are directly applicable to Cattle Egrets, which were one of the focal species in the study.

Although UAS and flush counts were faster and less labour‐intensive, ground nest counts and flight‐line surveys required considerably more time and effort. These methodological discrepancies complicate comparisons across studies and highlight the need for consistent, standardised protocols in long‐term monitoring. For Southern Africa, this is particularly important because historical ground‐based counts may not be directly comparable to modern aerial or UAS‐based surveys without adjusting for method‐specific biases. Ensuring methodological consistency or, at minimum, transparent correction factors is essential for producing reliable population trends and informing conservation planning (Prosser et al. 2022).

Cattle Egrets exhibit broad habitat flexibility, exploiting wetlands, grasslands, agricultural fields, and urban environments. In Tanzania, their distribution correlated positively with wetland and grassland availability (Kioko et al. 2016), while in South Africa, flock size was strongly associated with ostrich herd size (Kamler et al. 2008). Urban and agricultural landscapes provide abundant foraging opportunities. In Udaipur, India, the species used 29 roosts and 62 nest sites, within wetlands and built‐up areas (Mehta et al. 2024). In Australia, their extensive use of rice fields, where up to 60% of individuals forage, further illustrating their adaptability (Richardson and Taylor 2003).

### 3.2 Habitat Use and Foraging Ecology

Cattle Egrets in Southern Africa exhibit exceptional ecological plasticity, enabling them to exploit a wide range of natural and human‐modified habitats. Across the region, they occupy wetlands such as the Okavango Delta (Botswana), the Barotse Floodplain (though no primary data exist for Zambia), Chókwè irrigation canals in Mozambique, and estuarine systems including Durban Bay (South Africa). Their use of pastoral rangelands, communal grazing areas, cropped Highveld fields, irrigated plantations, and peri‐urban landfill sites further illustrates their ability to integrate into agricultural and urban mosaics (Cele et al. 2025; Louw 2005; Kopij 2017; Siegfried 1970, 1971a; Tarboton et al. 1987). Movements between wetlands, agricultural fields, and peri‐urban areas are common and driven by prey availability, hydrological conditions, and disturbance regimes, although no study has quantified the proportion of time spent in each habitat type.

Seasonal habitat shifts are described qualitatively across studies. During the dry season, Cattle Egrets concentrate around permanent water bodies such as Lake Chivero, Manyame Dams (Zimbabwe), the Vaal River system (South Africa), and Okavango floodplain channels (Botswana), where aquatic invertebrates and small fish accumulate (Hafner et al. 1986; Kopij and Paxton 2018). In the wet season, they move into ephemeral grasslands and floodplains such as Save Valley (Zimbabwe) and the Molopo plains (South Africa‐Botswana), where herbivore activity flushes Orthoptera, Coleoptera, and small vertebrates (Hering et al. 2017; Rashed 2015; Tarboton et al. 1987). Although some studies report percentages of habitat use, these values represent proportions of observations within individual studies rather than movements of the same individuals across habitats.

Nesting and roosting occur primarily in trees, often over or near water, providing protection from predators and disturbance. In Southern Africa, communal roosts typically contain 1000–2000 birds and may reach 5000 individuals (Siegfried 1971a, 1972b). Foraging distances usually remain within 19 km of the roost, with maximum recorded distances of 29 km, forming networks of roosts spaced approximately 16 km apart (Siegfried 1970, 1971a). Similar arboreal nesting has been documented in Algeria, Iran, and India (Ashoori and Barati 2013; Mehta et al. 2024; Metallaoui et al. 2020). Recent urban studies in KwaZulu Natal show that all colonies occur within 10 km of wetlands, rivers, grasslands, major roads, and landfills, highlighting the species' reliance on urban mosaic landscapes (Cele et al. 2025).

Cattle Egret foraging ecology is strongly shaped by their association with large mammals and human activities. By following cattle, buffalo, elephants, tractors, and human workers, they exploit prey flushed by movement, achieving capture rates 3.6–5.2 times higher when associated with cattle (Kioko et al. 2016). Their diet is primarily insectivorous, dominated by grasshoppers, locusts, caterpillars, crickets, moths, spiders, and centipedes (Siegfried 1971c, 1972a). A comparative summary of recorded prey types across regions is provided in Table 2. They also consume amphibians, reptiles, and small mammals, with similar patterns reported in Pakistan, India, and Australia (Malik and Zeb 2009; Seedikkoya and Azeez 2009; Stafford et al. 2010). Cattle Egrets frequently exploit anthropogenic disturbances, foraging behind agricultural machinery, grass‐cutting teams, and at waste dumps, slaughterhouses, and fish‐drying sites (Agunbiade et al. 2025; Yasmeen et al. 2019). Although primarily terrestrial, they also forage arboreally when prey availability is high, including feeding on pollinating insects and termite alates from elevated perches (Kushlan 1979; Platt et al. 2021).

**TABLE 2:** Extracted dietary categories and prey items recorded for Cattle Egrets across Southern Africa and comparative regions, illustrating broad trophic flexibility.

Prey type	Southern Africa (Siegfried 1972a, 1972b; Skead 1952, 1966)	Comparative Regions (Malik and Zeb 2009; Seedikkoya and Azeez 2009; Stafford et al. 2010; Telfair 1981; Zaman et al. 2022)
Insects	Grasshoppers, locusts, caterpillars, crickets, moths, spiders, centipedes	Moths, beetles, prawns, flies, snails, dragonflies, black beetles, maggots, waste rice, natural seeds, other invertebrates
Vertebrates	Frogs, toads, snakes, lizards, small mammals (mice), birds	Small lizards, frogs, small mammals, fish (less common)
Other	Earthworms	Earthworms

### 3.3 Breeding Ecology and Behaviour

Cattle Egrets employ flexible and opportunistic breeding strategies characterised by colonial nesting, frequent participation in mixed‐species aggregations, and reproductive output shaped primarily by food availability and rainfall‐driven pulses in prey abundance (Metallaoui et al. 2020; Bowker and Downs 2012). Across most of Southern Africa, breeding aligns with the spring–summer rainfall period, when invertebrate prey peak. In the Eastern Cape, breeding spans September to February (Skead 1952), whereas in the Western Cape—a winter‐rainfall Mediterranean system—breeding occurs from August to December, beginning shortly after the winter rainfall peak and extending into early summer as prey availability increases (Siegfried 1971a). In KwaZulu‐Natal and Mpumalanga, clutch initiation typically occurs within two weeks of the onset of summer rains, reflecting tight coupling between rainfall, prey emergence and breeding timing (Bowker and Downs 2012).

Breeding occurs in dense colonies that often include other ardeids, such as the Grey Heron ( *Ardea cinerea* ), Little Egret ( *Egretta garzetta* ), African Sacred Ibis ( *Threskiornis aethiopicus* ), and Glossy Ibis ( *Plegadis falcinellus* ) (Cele et al. 2025; Craig et al. 2021). Colony formation is strongly influenced by social cues, with individuals preferring to join established groups rather than initiating solitary nesting. This behaviour mirrors patterns observed internationally, where the abandonment of native heron colonies can lead to subsequent desertion by Cattle Egrets despite adequate terrestrial prey (Belzer and Lombardi 1989). Colony sizes in Southern Africa range from small monospecific groups of 12–48 nests in Lesotho (Kopij 1999) to mixed‐species heronries exceeding 500 nests in KwaZulu‐Natal and Mpumalanga (Burger 1978). A comparative overview of breeding characteristics across regions is presented in Table 3.

**TABLE 3:** Comparative overview of Cattle Egret breeding characteristics, including timing, nest placement, colony size, and species composition, based on studies from Southern Africa and international sites.

Location	Breeding season	Nest substrate	Colony size (Cattle Egret nests)	Mixed‐species colony
King William's Town, SA (Skead 1952, 1966)	September–February	Eucalyptus trees (40–60 ft. high)	429 (1949), 669 (1950); 2000+ birds	Yes (with Black‐headed Herons)
SW Cape Province, SA (Siegfried 1972b)	August–December	—	—	—
KwaZulu‐Natal, SA (Bowker and Downs 2012)	—	—	9 breeding events at 7 sites	Yes (41/47 Egret records)
KwaZulu‐Natal urban mosaic, SA (Cele et al. 2025)	October–January (inferred from breeding season monitoring)	*Vachellia*, *Eucalyptus*, *Jacaranda* spp.; trees near wetlands, landfills, WWTWs	Roosts: 500–600 birds; Nesting colonies present but nest counts not reported	Yes (mixed ardeid roosts)
Makhanda urban area, SA (Craig et al. 2021)	—	Urban trees, school grounds, gardens (breeding confirmed but no nest metrics)	—	Yes (urban assemblage of 104 breeding species)
Guerbes‐Sanhadja, Algeria (Metallaoui et al. 2020)	Mid‐April to late July	*Salix alba* , *Acacia cyanophylla* (avg 1.51 m high)	~750 pairs (2014)	—
Pelee Island, Canada (Putnam 1978)	Late Spring/Summer	Button bush (3–10 ft. high)	15–20 nests (1976)	Yes (with Black‐crowned Night Herons, Green Herons, Great Blue Herons, Common Egrets)
South Dakota, USA (Naugle et al. 1996)	—	····− (lowest stratum)	5680 (95% CI ± 1059)	Yes (95% Cattle Egret, Great Egret, Black‐crowned Night‐Heron)
Guyana (Lowe‐McConnell 1967)	April‐August, November‐December (two peaks)	Mid‐height bushes	1597 (August), 1700+ (December)	Yes (with indigenous herons)

Nest site selection consistently favours substrates within 20 m of permanent water, with mean nest heights of 2.3 m (±0.4 m) in Southern Africa (Cele et al. 2025; Louw 2005). Cattle Egrets nest in a wide range of environments, including reedbeds, mangroves, exotic plantations, peri‐urban trees, and occasionally on the ground near farm dams (Siegfried 1971a). Both sexes participate in nest construction, with males typically collecting sticks and females arranging them on the platform, a pattern consistent with other ardeids (Siegfried 1971a). However, nest loss during the building phase is common, largely due to theft of nesting material by conspecific species and other heron species (Siegfried 1972b).

Following pairing and nest construction, females typically lay clutches of 3 to 5 eggs. In the Western Cape (~33 S–34° S), early‐season clutches average 2.84–2.99 eggs, with numbers declining later in the season (Siegfried 1971b). International comparisons reveal a latitudinal pattern reflecting variations in day length and seasonal resource availability: larger clutches are observed at higher latitudes such as Algeria (3.43 eggs; ~36° N) and Iran (3.95 eggs; ~32 N–36° N), whereas smaller clutches (1–3 eggs) are typical in tropical Guyana (~6° N) where seasonal variation in day length and prey availability is minimal (Lowe‐McConnell 1967). Incubation lasts approximately 23 days, with both parents participating in the process (Siegfried 1971a).

Upon hatching, parental care includes brooding, shading, and frequent provisioning, culminating in a fledgling period of 30–35 days. Chick mortality is highest during the first 2 weeks post‐hatching, primarily due to the starvation of the last‐hatched chicks (Weber 1975). While sibling aggression occurs, it is not a major mortality factor in Southern Africa (Siegfried 1970).

Overall breeding success in the Western Cape averaged 24%–31%, yielding 0.54 fledged young per adult per season (Siegfried 1972b). Hatching success in the region (76%–83%) is relatively low compared to values reported in Iran (83%) but slightly higher than in Algeria (73.6%). Causes of reproductive failure include nest collapse, material theft, brood parasitism (González‐Martín and Ruiz 1996), starvation, predation by otters and mongooses (Siegfried 1965), and severe weather events such as cyclones and hailstorms (Porio 2011). Additionally, environmental contaminants such as lead, cadmium, and persistent organic pollutants (POPs) can impair reproduction by reducing hatching success and causing developmental abnormalities (Abdullah et al. 2015).

### 3.4 Conservation Challenges, Ecosystem Services and Management Implications

Although the Cattle Egret is one of the world's most adaptable and rapidly expanding ardeids, its populations in Southern Africa face a suite of anthropogenic and environmental pressures that operate at both landscape and population scales. At the same time, the species provides valuable ecosystem services, linking agricultural, wetland, and peri‐urban systems through its movement and foraging behaviour.

Human disturbance at breeding colonies can trigger nest abandonment and increase vulnerability to nest robbing, particularly when intrusive survey methods are employed (Prosser et al. 2022; Siegfried 1971b, 1972a). In KwaZulu‐Natal, the removal of roost trees during the breeding season destroyed more than 130 nests, illustrating the sensitivity of urban colonies to unregulated vegetation clearing (Cele et al. 2025). Human presence also alters foraging behaviour, with feeding group sizes decreasing near roads in newly colonised landscapes (Si Bachir et al. 2012).

Habitat alteration further shapes population dynamics. In south‐eastern Australia, particularly within the Murray‐Darling basin of New South Wales and Victoria, wetland loss driven by irrigation expansion and river regulation has reduced natural foraging habitats, pushing Cattle Egrets into agricultural landscapes where pesticide exposure becomes an associated risk (Richardson and Taylor 2003). In North America, large‐scale drainage of shallow lakes in the Midwest disrupted aquatic prey for native herons, leading to colony abandonment; the loss of these social cues subsequently led Cattle Egrets to desert the same sites despite adequate terrestrial prey (Belzer and Lombardi 1989). Long‐term colony use can also degrade habitat internally, with tree mortality reaching 78%–100% in quadrats used for more than three consecutive seasons (Belzer and Lombardi 1989).

Food scarcity during late‐summer dry seasons disproportionately affects juveniles, whose stomach contents contain only 78% of adult quantities during seasonal bottlenecks (Siegfried 1971a, 1972a). Starvation accounts for at least 60% of chick deaths, and extreme scarcity intensifies aggression, with dominant adults gaining preferential access to food (Woolfenden et al. 1976). Competition for nesting resources, including nest robbing and aggressive interactions, further contributes to nest failure (Siegfried 1971c; Metallaoui et al. 2020). Predation is generally a secondary threat but can be locally significant; in South Africa, Clawless Otters, Water Mongooses, and genets accounted for 25% of chick disappearances (Siegfried 1965, 1972b). Severe weather events including tropical cyclones, hailstorms, and rapid flood pulses cause colony collapses, nest loss, and reduced fledging success (Siegfried 1971b; Porio 2011). Climate‐linked disturbances are projected to intensify across Southern Africa, with complete loss of entire breeding cohorts already recorded in some seasons (Bowker and Downs 2012).

Beyond direct disturbance and habitat loss, environmental contamination represents a pervasive, population‐level threat across much of the species' range. Studies from Pakistan and Iran report exceptionally high concentrations of heavy metals and persistent organic pollutants (POPs) in Cattle Egret tissues, often exceeding thresholds associated with reproductive impairment and physiological stress (Abdullah et al. 2015; Malik and Zeb 2009; Yasmeen et al. 2019). Feather concentrations reached 297 μg/g Pb and 41 μg/g Cd, among the highest recorded globally (Abdullah et al. 2015). These pollutants originate from industrial effluent, municipal waste, agricultural runoff, and vehicle emissions. Concentrations and associated ecological impacts across regions are summarised in Table 4. Urban individuals consistently exhibit higher contaminant loads than rural birds, reflecting contact with wastewater treatment facilities, landfills, and peri‐urban livestock systems (Cele et al. 2025). Disease risks compound these pressures: Cattle Egrets can harbour antibiotic‐resistant bacteria, such as resistant Micrococcus spp. in Nigeria (Fasina et al. 2014), and haemosporidian parasites are widespread, with a 25.5% Plasmodium prevalence across African populations (Villar Couto et al. 2019). Although a Zimbabwean study found no evidence of avian influenza infection (Caron et al. 2011), continental reviews report AIV prevalence of 3%–5% in terrestrial waterbirds frequenting domestic sites (Fuller et al. 2015; Gaidet et al. 2012).

**TABLE 4:** Summary of heavy metal contaminants recorded in Cattle Egret tissues across global studies, showing tissue types affected, concentration ranges, and associated ecological or physiological impacts.

Contaminant	Tissue	Southern Africa	Comparative regions (e.g., Pakistan, Iran)	Potential impact
Lead (Pb)	Feathers, blood, bone, muscle	—	37.5–76.5 μg/g (feathers) (Malik and Zeb 2009), 0.46 μg/g (blood) (Zaman et al. 2022), 47.32 μg/g (bone), 52.86 μg/g (muscle), 297 μg/g (feathers, industrial) (Abdullah et al. 2015)	Adverse effects, reproductive impairment (Abdullah et al. 2015; Malik and Zeb 2009)
Cadmium (Cd)	Feathers, liver	—	2.4–3.1 μg/g (feathers) (Malik and Zeb 2009), 0.46 μg/g (liver) (Zaman et al. 2022), 1.41 μg/g (liver, rural) (Yasmeen et al. 2019), 41 μg/g (feathers, industrial) (Abdullah et al. 2015)	Adverse effects, reproductive impairment (Abdullah et al. 2015; Malik and Zeb 2009)
Chromium (Cr)	Pectoral muscle, feathers	—	0.17 μg/g (pectoral muscle) (Zaman et al. 2022), 21.1 μg/g (feathers, industrial) (Abdullah et al. 2015)	Adverse effects, reproductive impairment (Abdullah et al. 2015; Malik and Zeb 2009)
Mercury (Hg)	Feathers, liver, kidney, muscle	—	1.6 μg/g (feathers), ~1.0 μg/g (liver) (Zamani‐Ahmadmahmoodi et al. 2010)	Lower levels in Cattle Egrets compared to aquatic feeders due to diet (Zamani‐Ahmadmahmoodi et al. 2010)

Despite these pressures, Cattle Egrets provide several important ecosystem services that benefit agricultural, wetland, and urban systems. Their foraging behaviour underpins natural pest suppression, with documented feeding on Army Worm outbreaks and consumption of 175–200 g of maggots per day at fish drying sites (Skead 1952; Seedikkoya and Azeez 2009). These services can reduce the need for chemical pesticides and align with precision agriculture goals of lowering chemical inputs (Padhiary et al. 2025). Nutrient cycling is another key service: guano deposition beneath communal roosts increases soil nitrogen and phosphorus by 12%–15% in Zimbabwe and Limpopo (Markwell and Driessen 1990), and movements between wetlands and agricultural fields facilitate seed dispersal, with more than 25 plant species dispersed via endozoochory and mean dispersal distances of approximately 8 km (Green and Elmberg 2013). Waterbird assemblages can supply up to 50% of carbon and 70% of phosphorus inputs in nutrient‐poor wetlands (Boros, Mozsár, et al. 2008; Boros, Várbíró, and Lengyel 2008), illustrating the scale of nutrient transfer possible in systems heavily used by Cattle Egrets. Large waterbird colonies can, however, also alter nutrient and trace element dynamics in surrounding waters: guano deposition elevates concentrations of potassium, calcium, iron, zinc, and copper, with zinc levels exceeding United States Environmental Protection Agency (US EPA) criteria in some systems (Telesford‐Checkley et al. 2016; Telesford‐Checkley, Mora, Grant, et al. 2017; Telesford‐Checkley, Mora, Gentry, et al. 2017), illustrating the complex role of Egrets as both beneficiaries and drivers of environmental change.

Urban ecosystem roles are increasingly important. In Makhanda (South Africa), Cattle Egrets form part of a diverse urban bird assemblage, using school grounds, sports fields, commonage, and suburban green spaces for foraging and roosting (Craig et al. 2021). These patterns highlight the importance of urban green infrastructure, mature trees, parks, and institutional grounds in sustaining waterbird presence within expanding towns and cities and underscore the need to integrate Cattle Egret habitat requirements into urban planning and conservation frameworks.

### 3.5 Conservation Implications and Management Strategies

The conservation of Cattle Egrets, particularly in Southern Africa, requires a multi‐faceted approach addressing both threats and leveraging their ecological benefits. Protecting urban roost trees and regulating vegetation clearing around wastewater treatment works and landfills are critical for maintaining colony persistence in mosaic landscapes (Cele et al. 2025).

### 3.6 Monitoring and Survey Methods

Accurate population monitoring is fundamental for conservation. The choice of survey method significantly impacts estimates, with UAS counts and flush counts producing significantly lower estimates than nest counts and flight‐line surveys (Prosser et al. 2022). Less intrusive methods like small unmanned aerial systems (UAS) are recommended to minimise disturbance to breeding colonies, especially given that disturbance itself is a threat (Prosser et al. 2022).

### 3.7 Habitat Management

Protecting and managing key habitats, particularly wetlands and large trees for roosting and nesting, is crucial. In urbanising landscapes, the retention of large, mature trees and the preservation of wetlands are vital for supporting waterbird populations (Mehta et al. 2024). Effective waste management practices and improved fencing at anthropogenic food sources (e.g., slaughterhouses) can reduce interspecific competition and mitigate disease risks (Agunbiade et al. 2025). Sustainable agricultural practices also played a central role. Precision‐agriculture systems, including AI‐driven irrigation scheduling and targeted chemical application, reduced water extraction and chemical runoff by 20%–40% and improved water‐use efficiency by up to 50% (Padhiary et al. 2025). These reductions directly benefited wetland integrity and lowered contaminant exposure for waterbirds, reinforcing the value of integrating Cattle Egret‐friendly agroecological practices, such as maintaining wet field margins and reducing pesticide use with emerging precision‐farming technologies.

### 3.8 Pollution Control and Sustainable Agriculture

Given the widespread threat of contaminants, stringent pollution control measures are essential. Padhiary et al. (2025) pointed out that industrial and agricultural pollutants, particularly heavy metals and persistent organic pollutants, posed chronic risks to reproductive success. Precision‐agriculture approaches offered a complementary mitigation pathway: AIguided fertiliser and pesticide optimisation reduced chemical inputs by 30%–40% and lowered CO_2_ emissions by approximately 20% (Padhiary et al. 2025). Encouraging natural pest control services provided by Cattle Egrets alongside precision‐agriculture technologies, offer a sustainable alternative to hazardous chemical use and reduced the ecological footprint of farming systems (Padhiary et al. 2025; Seedikkoya and Azeez 2009).

### 3.9 Understanding Ecological Interactions

Further research into the complex ecological interactions of Cattle Egrets is needed. For instance, understanding the impact of declining large mammal populations on Egret foraging success in savanna ecosystems (Kioko et al. 2016) or the role of interspecific social facilitation in colony establishment (Belzer and Lombardi 1989) can inform broader conservation strategies. The long‐standing ‘tick‐eating controversy’ illustrates how easily the species' ecological role can be mischaracterised. Early accounts portrayed Cattle Egrets as major tick‐removers from livestock, but quantitative studies showed that ticks form only a negligible proportion of their diet (Siegfried 1971b, 1972a), which is dominated by orthopterans and other mobile insects flushed by grazing mammals (Skead 1952, 1966). This misconception persists in popular and agricultural narratives, highlighting the need for accurate ecological communication to avoid overstating their role in parasite control and to ensure that conservation messaging reflects empirical evidence.

### 3.10 Regional Differences and Adaptability

The Cattle Egret's remarkable adaptability is evident in its rapid colonisation of new areas (Tarboton et al. 1987; Taylor et al. 2019; Underhill et al. 2016), its utilisation of human‐modified habitats like rice fields (Richardson and Taylor 2003) and urban environments (Cele et al. 2025; Craig et al. 2021; Mehta et al. 2024; Si Bachir et al. 2012), and its varied foraging associations (Burger and Gochfeld 1989; Kamler et al. 2008). However, this adaptability also means that threats and conservation priorities can vary regionally. Latitudinal differences in reproductive output, for example, smaller clutches in tropical populations compared to larger clutches in temperate Southern Africa, illustrate how local environmental conditions shape life‐history strategies (Lowe‐McConnell 1967). Yet the core threats of pollution, disturbance, and food scarcity remain globally relevant (Lesch, Pieters, and Bouwman 2024; Lesch, Kylin, and Bouwman 2024).

In conclusion, while the Cattle Egret is a resilient and successful coloniser, its populations, particularly in Southern Africa, are not immune to anthropogenic pressures (Cele et al. 2025; Craig et al. 2021). A comprehensive conservation strategy integrating robust monitoring, targeted habitat management, pollution control, and a nuanced understanding of ecological interactions would be essential for sustaining the species across rapidly changing socio‐ecological landscapes.

## 4 Discussion

This review has shown the Cattle Egret as a species whose ecological success in Southern Africa is underpinned by behavioural plasticity, broad habitat tolerance, and the ability to exploit human‐modified landscapes. Across the four thematic areas examined population status, habitat use and foraging ecology, breeding ecology, and threats and ecosystem services a consistent pattern emerges: the species thrives where productivity is high, disturbance is predictable, and anthropogenic structures create novel foraging and roosting opportunities. Yet this apparent resilience masks substantial knowledge gaps, uneven research effort, and emerging pressures that could reshape regional population trajectories.

**FIGURE 3:**
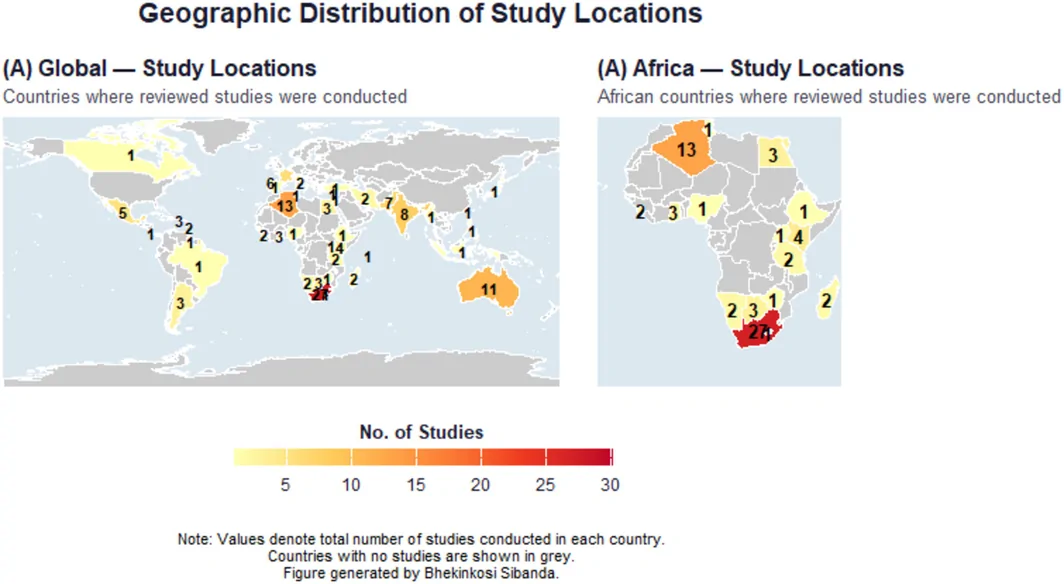
Global and African distribution of study locations. Shading and labels denote the number of studies conducted per country. Global or region‐wide reviews lacking country‐specific locations are not mapped.

Population trends across Southern Africa were generally stable or increasing, consistent with global patterns of rapid colonisation and expansion. Atlas‐derived reporting rates, long‐term roost counts, and colony‐level studies all point to sustained growth in well‐surveyed regions such as KwaZulu‐Natal, Mpumalanga, and the Eastern Cape. These increases align with the species' capacity to exploit irrigated agriculture, peri‐urban green spaces, and livestock‐rich rangelands habitats that have expanded markedly over the past five decades. However, this apparent stability is geographically uneven (Figure 3). Entire countries with extensive wetland systems, notably Zambia, lack primary ecological data, creating blind spots in regional assessments. These gaps mirror broader patterns in African ornithology where research is concentrated in a handful of countries and urban centres (Craig et al. 2021), meaning that regional population resilience may be overestimated and localised declines may go undetected (Figure 3).

The species' success is closely tied to its ability to exploit heterogeneous, human‐dominated landscapes. Across Southern Africa, Cattle Egrets moved fluidly between wetlands, agricultural fields, communal rangelands, and peri‐urban environments, tracking prey availability and hydrological cycles. Their foraging ecology centred on disturbance‐flushed prey makes them particularly responsive to livestock movements, mechanical harvesting, and urban waste streams. Urban studies from KwaZulu‐Natal and the Eastern Cape further demonstrate that Cattle Egrets are increasingly embedded within city‐scale ecological networks, with roosts and nesting colonies consistently occurring near landfills, wastewater treatment works, and managed green spaces (Cele et al. 2025; Craig et al. 2021). These findings suggest that urbanisation, often detrimental to biodiversity, may create novel ecological opportunities for disturbance‐adapted waterbirds.

Breeding patterns reflected a combination of hydrological cues, substrate availability, and social facilitation, with colonies typically initiated shortly after the onset of seasonal rains. Mixed‐species colonies were common, and social cues from other ardeids likely facilitated colony establishment, mirroring patterns observed in North America and the Caribbean. Breeding success, however, varied widely: South African studies reported relatively low hatching success and high early‐stage chick mortality driven primarily by starvation of last‐hatched chicks, while studies from Algeria and Iran documented substantially higher reproductive success, suggesting that local food availability, predation pressure, and disturbance regimes strongly mediate breeding outcomes.

Cattle Egrets occupy a paradoxical position at the wildlife–livestock–human interface. On one hand, they provide valuable ecosystem services including natural pest suppression, nutrient redistribution through guano deposition, and seed dispersal across wetland–savanna mosaics, services that align with precision agriculture goals of reducing chemical inputs (Padhiary et al. 2025). On the other hand, the same behaviours that generate these services expose the species to contaminants, pathogens, and human–wildlife conflict. Urban and peri‐urban colonies experience elevated exposure to heavy metals and antibiotic‐resistant bacteria, roost trees are frequently removed during breeding seasons, and extreme weather events have caused complete breeding failures in some years. These pressures highlight the species' vulnerability despite its generalist ecology.

Taken together, the evidence suggests that the Cattle Egret's regional success is tightly coupled to socio‐ecological transformations across Southern Africa. Agricultural intensification, urban expansion, and hydrological modification have created new opportunities, but have also introduced novel threats. The species' future trajectory will depend on how these opposing forces evolve and underscore the need for integrated, cross‐disciplinary approaches that link waterbird ecology with agricultural policy, urban planning, hydrological management, and One Health frameworks. Only through such integration can the dual role of the Cattle Egret as both a beneficiary and a sentinel of environmental change be fully understood and effectively managed.

### 4.1 Limitations

This review is constrained by several interrelated limitations. The available literature is geographically uneven, with research effort concentrated in South Africa and Zimbabwe and sparse coverage elsewhere; no primary ecological studies were found for Zambia despite its extensive Ramsar wetlands. Methodological diversity across studies, including differences in survey effort, sampling design, and analytical approaches limits comparability, as does the inclusion of grey literature, which introduces variability in reporting quality. Few studies employed advanced tracking or isotope techniques, restricting inference on movement ecology and trophic pathways. Thematically, habitat and foraging ecology dominated the evidence base (43.9% of studies), while breeding biology, threats, and ecosystem services remained under‐represented. Taken together, these gaps constrain region‐wide conclusions and highlight the need for more standardised, thematically balanced research across the subcontinent.

## 5 Conclusion

This review synthesises nearly eight decades of research on Cattle Egrets in Southern Africa, highlighting the species' dual role as a resilient generalist thriving in productive, human‐modified landscapes and as a vulnerable component of biodiversity in regions where ecological poor or degraded regions remain sparse. Its capacity to exploit agricultural, wetland, and peri‐urban mosaics underpins key ecosystem services, including natural pest suppression, nutrient redistribution, and seed dispersal, while its sensitivity to contaminants and pathogens positions it as an important sentinel at the wildlife–livestock–human interface.

Despite these strengths, the evidence base, however, remains uneven. Geographic gaps, particularly in countries such as Zambia and Angola, and methodological inconsistencies across decades of monitoring constrain our ability to generate robust regional population trends. Addressing these limitations will require a coordinated, standardised monitoring framework and the integration of modern tools capable of resolving movement ecology, trophic dynamics and colony distribution at appropriate spatial scales. Aligning future research with these gaps will strengthen conservation planning and improve the capacity of managers to respond to emerging socio‐ecological pressures, including hydrological alteration, agrochemical exposure, and climate‐linked habitat change.

## 6 Recommendations

Securing the ecological and socio‐economic benefits provided by Cattle Egrets requires coordinated, multi‐level action. Priority measures include protecting roosting and nesting trees, maintaining natural hydrological regimes in Ramsar and other key wetlands, and promoting egret‐friendly agroecological practices such as preserving wet field margins and reducing pesticide use. In intensifying agricultural landscapes, precision‐agriculture approaches that reduce chemical inputs by 20%–40% would mitigate exposure risks while enhancing the species' ecosystem‐service contributions.

Future research should prioritise advanced tracking technologies such as GPS telemetry, automated radio‐tracking, and stable isotope analyses to resolve fine‐scale movements, seasonal connectivity, and trophic pathways. Expanding research into under‐studied countries will correct the pronounced geographic bias, while targeted studies on breeding ecology, demographic rates, and colony dynamics will fill the thematic gaps highlighted in this review. Embedding Cattle Egrets within One Health surveillance systems will improve early detection of zoonotic threats, particularly as the species increasingly occupies peri‐urban and agricultural interfaces.

Strengthening flyway‐level cooperation under the Agreement on the Conservation of African‐Eurasian Migratory Waterbirds (AEWA) will support transboundary monitoring and adaptive management. Enhanced atlas updates, citizen‐science participation, and standardised survey methods across countries will improve spatial coverage and reduce inconsistencies that currently limit regional synthesis. Integrating insights from climate‐vulnerability research, mining‐related ecological degradation, precision‐agriculture transitions, and urban bird ecology will situate Cattle Egret ecology within broader socio‐ecological change across the subcontinent. Integrating Cattle Egrets into One Health surveillance has direct implications for managing disease risks at the wildlife–livestock–human interface. Because the species routinely moves between wetlands, rangelands, peri‐urban waste sites, and livestock facilities, it can act as an early‐warning sentinel for pathogens circulating across these systems. A One Health approach enables coordinated monitoring of avian influenza viruses, enteric bacteria, parasites, and contaminants by linking data from wildlife populations (e.g., colony health, pathogen prevalence), livestock operations (e.g., poultry outbreaks, biosecurity breaches), and human health systems (e.g., zoonotic spillover events). For Cattle Egrets, this integration supports targeted surveillance at high‐risk contact points such as cattle kraals, poultry farms, abattoirs, and landfill sites, improves detection of emerging pathogens, and informs management actions including habitat sanitation, livestock biosecurity, and wetland health assessments. By embedding the species within a cross‐sectoral monitoring framework, One Health strengthens both conservation outcomes and public‐health preparedness.

Policymakers, conservation agencies, and research institutions should implement these measures now. Each recommended action is grounded in the limitations identified in this review, ensuring that interventions are targeted, evidence‐based, and responsive to the most pressing knowledge and conservation gaps.

## Author Contributions

**Bhekinkosi Sibanda:** conceptualization (lead), formal analysis (lead), methodology (equal), writing – original draft (lead). **Tawanda Tarakini:** conceptualization (equal). **Josphine Mundava:** conceptualization (equal).

## Funding

This research was funded by Oppenheimer Generations Research and Conservation and Shangani Holistic (Pvt) Ltd. (MOA 03/2024/001) and is hosted by the Department of Wildlife Ecology and Conservation, CUT, under the coordination of the ISANGO Project (CIRAD).

## Disclosure

Permission to Reproduce Material: All figures and tables are original to this study. No previously published material has been reproduced.

## Ethics Statement

This article is a systematic review and meta‐analysis of previously published literature and did not involve new data collection from animals or humans; therefore, no ethics approval was required.

## Conflicts of Interest

The authors declare no conflicts of interest.

## Supporting information


**Data S1:** Detailed search strategy.
**Data S2:** PRISMA flow summary.
**Data S3:** Studies used in this review.
**Data S4:** Master study metadata table.
**Data S5:** Analytical code snippets (R Pseudocode).

## Data Availability

All data supporting the findings of this review are contained within the article and its Supporting Information. Additional bibliometric datasets and extraction tables are available from the corresponding author upon reasonable request.
