# Supplementary material for: Population Status, Ecology and Conservation Challenges of Cattle Egrets (Bubulcus ibis), Especially in the Southern Africa Subregion: A Systematic Review and Evidence Synthesis
Source: Ecol Evol. 2026 Sep 15;16(9):e74300. doi: 10.1002/ece3.74300 (PMC13575867; doi:10.1002/ece3.74300)
Supplement: Supplementary file 1 [file ECE3-16-e74300-s001.docx]

**Supplementary Information**

**S1. Detailed Search Strategy**

Databases searched

- Scopus (Elsevier)
- Web of Science Core Collection (Clarivate)

Search period

- Searches conducted between July 2025 and March 2026
- No lower date limit applied
- Earliest retrieved record: 1952

Search scope & syntax

- Combined scientific and common names of *Bubulcus ibis* with ecological, behavioural and conservation terms.
- Boolean operators (AND, OR) and wildcards (*) adapted for each database.
- English‐language filter in Scopus; all languages retained in Web of Science.
- Search strings followed ROSES and PRISMA 2020 guidance

Exact search strings

Scopus

TITLE-ABS-KEY("Bubulcus ibis" OR "cattle egret*")

AND TITLE-ABS-KEY(population OR distribution OR abundance OR "range expansion" OR migration OR dispersal OR "habitat use" OR foraging OR diet OR breeding OR reproduction OR nesting OR "colony size" OR roost* OR "land use" OR agriculture OR "rice field*" OR wetland* OR "threat*" OR "ecosystem service*" OR conservation)

AND TITLE-ABS-KEY("Southern Africa" OR Angola OR Botswana OR Eswatini OR Lesotho OR Malawi OR Mozambique OR Namibia OR "South Africa" OR Zambia OR Zimbabwe)

LANGUAGE(English)

Web of Science Core Collection

TS=("Bubulcus ibis" OR "cattle egret*")

AND TS=(population OR distribution OR abundance OR "range expansion" OR migration OR dispersal OR "habitat use" OR foraging OR diet OR breeding OR reproduction OR nesting OR "colony size" OR roost* OR "land use" OR agriculture OR "rice field*" OR wetland* OR "threat*" OR "ecosystem service*" OR conservation)

AND TS=("Southern Africa" OR Angola OR Botswana OR Eswatini OR Lesotho OR Malawi OR Mozambique OR Namibia OR "South Africa" OR Zambia OR Zimbabwe)

*(TS = Topic search across title, abstract, author keywords, Keywords Plus®)*

Reference management, deduplication & screening

- All records exported from Scopus and Web of Science.
- Imported into RStudio for deduplication using *bibliometrix.*
- Deduplicated based on DOI, title, authors and year.
- Screening (title → abstract → full text) conducted in R and Excel.
- Citations chaining used to identify additional eligible studies.
- All screening decisions recorded following ROSES guidance.

**S2. PRISMA Flow Summary**

A total of 589 records were retrieved; after deduplication and screening, 173 studies were included in the final synthesis.

| Stage | Records | Excluded | Remaining | Reason for Exclusion |
| --- | --- | --- | --- | --- |
| Records identified | 589 | – | 589 | Database search |
| Duplicates removed | – | 31 | 558 | Exact title/author/year matches |
| Titles screened | 558 | 190 | 368 | Irrelevant to *B. ibis* or Southern Africa |
| Abstracts screened | 368 | 161 | 207 | Not primary/secondary ecological data |
| Full text sought | 207 | 34 | 173 | Full text unavailable |
| Full texts assessed | 173 | 0 | 173 | All eligible |
| Studies included in synthesis |  | – | 173 | Final synthesis |

**S3. Studies Used In This Review**

| **No.** | **Author(s)** | **Title** |
| --- | --- | --- |
| 1 | Abdullah, M., Khan, R. A., Rafay, M., Hussain, T., Ruby, T., Rehman, F., ... & Akhtar, S. (2017). | Habitat ecology and breeding performance of Cattle Egret (Bubulcus ibis) in Faisalabad, Pakistan. |
| 2 | Amissah, C., Iwajomo, S. B., Abraham, J., & Deikumah, J. P. (2024). | Urban lawn vegetation structure impact arthropod abundance and cattle egret foraging success. |
| 3 | Andiatsirevombola, J. M., Ge, J., Nyirenda, T. M., & Raholijaona, R. (2016). | Predation impact of cattle egret (Bubulcus ibis) on migratory locust (Locusta migratoria capito) and red locust (Nomadacris septemfasciata) in south and southwest regions of Madagascar. |
| 4 | Arendt, W. J. (1988). | Range expansion of the cattle egret (Bubulcus ibis) in the Greater Caribbean Basin. |
| 5 | Ashoori, A., & Barati, A. (2013). | Breeding success of Black-crowned Night Heron (Nycticorax nycticorax), Little Egret (Egretta garzetta) and Cattle Egret (Bubulcus ibis) (Aves: Ardeidae) in relation to nest height in the South Caspian Sea. |
| 6 | Ashoori, A., Moradi, H. V., Rezaiee, H. R., & Mahiny, A. S. (2017). | Dietary segregation of four ardeid species breeding in Anzali International Wetland, Northern Iran. |
| 7 | Axelsen, J. R. A., Petersen, B. S., Maiga, I. H., Niassy, A., Badji, K., Ouambama, Z., ... & Kooyman, C. (2009). | Simulation studies of Senegalese Grasshopper ecosystem interactions II: the role of egg pod predators and birds. |
| 8 | Bachir, A. S., Barbraud, C., Doumandji, S., & Hafner, H. (2008). | Nest site selection and breeding success in an expanding species, the Cattle Egret Bubulcus ibis. |
| 9 | Baker, N. J., & Dieter, C. D. (2015). | Reproductive success of colonial tree-nesting waterbirds in prairie pothole wetlands and rivers throughout northeastern South Dakota. |
| 10 | Baxter, G. S., & Fairweather, P. G. (1998). | Does available foraging area, location or colony character control the size of multispecies egret colonies?. |
| 11 | Baxter, G. S. (1994). | The location and status of egret colonies in coastal New South Wales. |
| 12 | Baxter, G. S., & Fairweather, P. G. (1989). | Comparison of the Diets of Nestling Cattle Egrets and Intermediate Egrets in the Hunter Valley, New-South-Wales. |
| 13 | Beauchamp, G. (1999). | The evolution of communal roosting in birds: origin and secondary losses. |
| 14 | Beckett, T. A., III. (1965). | Cattle Egrets Colour Banded. |
| 15 | Belozerov, V. N., Van Niekerk, D. J., & Butler, H. J. B. (2003). | Population structure of Argas arboreus (Acari: Argasidae) ticks associated with seasonally abandoned mixed heronries, dominated by cattle egrets (Bubulcus ibis), in South Africa. |
| 16 | Belzer, W. R., & Lombardi, J. R. (1989). | Cattle Egret Symbiosis and Heronry Abandonment. |
| 17 | Berthold, P. (1999). | A comprehensive theory for the evolution, control and adaptability of avian migration. |
| 18 | Boukhemza, M., Doumandji, S., Voisin, C., & Voisin, J. F. (2004). | Comparative utilization pattern of trophic resources by White storks Ciconia ciconia and Cattle egrets Bubulcus ibis in Kabylia (Algéria). |
| 19 | Bouwman, H., Yohannes, Y. B., Nakayama, S. M. M., Motohira, K., Ishizuka, M., Humphries, M. S., ... & Ikenaka, Y. (2019). | Evidence of impacts from DDT in pelican, cormorant, stork, and egret eggs from KwaZulu-Natal, South Africa. |
| 20 | Bouwman, H., Polder, A., Venter, B., & Skaare, J. U. (2008). | Organochlorine contaminants in cormorant, darter, egret, and ibis eggs from South Africa. |
| 21 | Bouwman, H., Viljoen, I. M., Quinn, L. P., & Polder, A. (2013). | Halogenated pollutants in terrestrial and aquatic bird eggs: converging patterns of pollutant profiles, and impacts and risks from high levels. |
| 22 | Bowker, M. B., & Downs, C. T. (2012). | Breeding of large, water-associated, colonially nesting birds of the north-eastern region of KwaZulu-Natal, South Africa. |
| 23 | Browder, J. A. (1973). | Long-distance movements of Cattle Egrets. |
| 24 | Burger, J., & Gochfeld, M. (1989). | Age differences in Cattle Egrets Bubulcus ibis, foraging with wild ungulates in Kenya. |
| 25 | Burger, J. (1978). | Competition Between Cattle Egrets and Native North American Herons, Egrets, and Ibises. |
| 26 | Burger, J., & Gochfeld, M. (1982). | Host selection as an adaptation to host-dependent foraging success in the cattle egret (Bubulcus ibis). |
| 27 | Burger, J., & Gochfeld, M. (1993). | Making foraging decisions: host selection by Cattle Egrets Bubulcus ibis. |
| 28 | Burger, J. (1982). | On the nesting location of cattle egrets (Bubulcus ibis) in South African heronries. |
| 29 | Burger, J., Parsons, K., Benson, T., Shukla, T., Rothstein, D., & Gochfeld, M. (1992). | Heavy metal and selenium levels in young cattle egrets from nesting colonies in the northeastern United States, Puerto Rico, and Egypt. |
| 30 | Burger, J., & Gochfeld, M. (1997). | Heavy metal and selenium concentrations in feathers of egrets from Bali and Sulawesi, Indonesia. |
| 31 | Burger, J., & Gochfeld, M. (1990). | Vertical nest stratification in a herony in Madagascar. |
| 32 | Caron, A., Grosbois, V., Etter, E., Gaidet, N., & de Garine-Wichatitsky, M. (2014). | Bridge hosts for avian influenza viruses at the wildlife/domestic interface: An eco-epidemiological framework implemented in southern Africa. |
| 33 | Cele, J. Z., Maseko, M. S. T., & Downs, C. T. (2025). | Western Cattle Egrets’ (Bubulcus ibis) roosting colony size estimates in urban landscape mosaics of KwaZulu-Natal, South Africa, with notes on habitat threats and features near roosts. |
| 34 | Champagnon, J., Carré, H., & Gili, L. (2019). | Effects of research disturbance on nest survival in a mixed colony of waterbirds. |
| 35 | Charalambidou, I., & Gucel, S. (2013). | First record of mixed-species heron colony: Cattle Egret (Bubulcus ibis), Squacco Heron (Ardeola ralloides) and Little Egret (Egretta garzetta) breeding at Famagusta freshwater lake in Cyprus. |
| 36 | Congrains, C., Carvalho, A. F., Miranda, E. A., Cumming, G. S., Henry, D. A., Manu, S. A., ... & Lama, S. N. D. (2016). | Genetic and paleomodelling evidence of the population expansion of the cattle egret Bubulcus ibis in Africa during the climatic oscillations of the Late Pleistocene. |
| 37 | Corn, J. L., Barré, N., Thiebot, B., Creekmore, T. E., Garris, G. I., & Nettles, V. F. (1993). | Potential role of cattle egrets, Bubulcus ibis (Ciconiformes: Ardeidae), in the dissemination of Amblyomma variegatum (Acari: Ixodidae) in the eastern Caribbean. |
| 38 | Craig, A. J. F. K., Hulley, P. E., & Mullins, R. L. G. (2021). | Urban birds in the Eastern Cape: Local observations from Makhanda (Grahamstown) and future questions. |
| 39 | Craufurd, R. Q. (1966). | Notes on the ecology of the Cattle Egret Ardeola ibis at Rokupr, Sierra Leone. |
| 40 | Dami, L., Bennetts, R. E., & Hafner, H. (2006). | Do Cattle Egrets exclude Little Egrets from settling at higher quality sites within mixed-species colonies?. |
| 41 | De La Paz Ducommun, M., Quiroga, M. A., Beltzer, A. H., & Schnack, J. A. (2009). | Diet of Cattle Egrets (Bubulcus ibis ibis) in the flood valley of the Paraná River, northern Argentina. |
| 42 | Dinsmore, J. J. (1973). | Foraging Success of Cattle Egrets, Bubulcus ibis. |
| 43 | Evans, K. L., Hatchwell, B. J., Parnell, M., & Gaston, K. J. (2010). | A conceptual framework for the colonisation of urban areas: The blackbird Turdus merula as a case study. |
| 44 | Faber, R. A., Risebrough, R. W., & Pratt, H. M. (1972). | Organochlorines and mercury in common egrets and great blue herons. |
| 45 | Feare, C. J. (1975). | Scavenging and kleptoparasitism as feeding methods of Seychelles Cattle Egrets Bubulcus ibis. |
| 46 | Fernandez, E. V., Shi, X., & Li, Z. (2017). | Ritualized fights among foraging cattle egrets following Père David's deer in Dafeng Nature Reserve. |
| 47 | Fogarty, M. J., & Hetrick, W. M. (1973). | Summer foods of Cattle Egrets in north central Florida. |
| 48 | Francis, R., Bino, G., Inman, V., Brandis, K., & Kingsford, R. T. (2021). | The Okavango Delta’s waterbirds–Trends and threatening processes. |
| 49 | Fujioka, M. (1985). | Food delivery and sibling competition in experimentally even-aged broods of the cattle egret. |
| 50 | Garrido, J. R., Sarasa, C. G., & Fernández-Cruz, M. (2002). | Intraspecific kleptoparasitism in the cattle egret. |
| 51 | Gassett, J. W., Folk, T. H., Alexy, K. J., Miller, K. V., Chapman, B. R., Boyd, F. L., & Hall, D. I. (2000). | Food habits of cattle egrets on St. Croix, US Virgin Islands. |
| 52 | Gbogbo, F., Acheampong, G. K., Atiemo, J. M. Y., & Crepindale, Q. E. (2013). | Habitat use pattern of three species of egrets in a small coastal lagoon in Ghana. |
| 53 | Gerhardt, F., & Taliaferro, E. H. (2003). | Density-dependent patch selection by foraging Cattle Egrets. |
| 54 | Getachew, M., Ambelu, A., Tiku, S., Legesse, W., Adugna, A., & Kloos, H. (2012). | Ecological assessment of Cheffa Wetland in the Borkena Valley, northeast Ethiopia: Macroinvertebrate and bird communities. |
| 55 | Gherbi-Salmi, R., Doumandji, S., & Voisin, C. (2012). | Diet of chicks of Cattle Egrets Bubulcus ibis in the lower Soummam valley, Algeria. |
| 56 | Goddard, M. T. (1955). | Notes on the breeding of the Cattle Egret in north-eastern New South Wales. |
| 57 | Gonzalez-Voyer, A., & Drummond, H. (2007). | Is broodmate aggression really associated with direct feeding?. |
| 58 | Green, A. J., & Elmberg, J. (2013). | Ecosystem services provided by waterbirds. |
| 59 | Grenfell, B. T., & Gulland, F. M. D. (1995). | Introduction: ecological impact of parasitism on wildlife host populations. |
| 60 | Hafner, H., Pineau, O., & Wallace, J. P. (1992). | The effects of winter climate on the size of the Cattle Egret (Bubulcus ibis L.) population in the Camargue. |
| 61 | Heatwole, H. (1965). | Some aspects of the association of Cattle Egrets with cattle. |
| 62 | Hering, J., Fischer, S., Geiter, O., Wobker, J., Siegel, S., Eilts, H.-J., Fuchs, E., Hoek, D., & Habib, M. (2020a). | Large breeding colonies of Herons, Egrets and Glossy Ibis at Lake Nasser (Egypt). |
| 63 | Hering, J., Fischer, S., Fuchs, E., Habib, M. I., & Wobker, J. (2020b). | Yellow-billed Storks at Lake Nasser and status in Egypt. |
| 64 | Hilaluddin, Shah, J. N., & Shawl, T. A. (2003). | Nest site selection and breeding success by cattle egret and little egret in Amroha, Uttar Pradesh, India. |
| 65 | Hudson, J. W., Dawson, W. R., & Hill, R. W. (1974). | Growth and development of temperature regulation in nestling cattle egrets. |
| 66 | Imam Fadila, M., Mardiastuti, A., & Aryati Mulyani, Y. (2020, July). | Insects as food source for cattle egret (Bubulcus ibis) in Jatibarang landfill, Semarang. |
| 67 | Jenni, D. A. (1973). | Regional variation in the food of Nestling Cattle Egrets. |
| 68 | John, J. R., & Lee, W. S. (2012). | First record of the association of Cattle Egret Bubulcus ibis with Shoebill Balaeniceps rex. |
| 69 | Kamler, J. F., Suinyuy, T. N., & Goulding, W. (2008). | Cattle Egret and Common Ostrich associations in South Africa. |
| 70 | Kersten, M., Britton, R. H., Dugan, P. J., & Hafner, H. (1991). | Flock feeding and food intake in little egrets: the effects of prey distribution and behaviour. |
| 71 | Khan, M., Mohammad, A., Ahad, K., Katsoyiannis, A., Malik, S. A., Abdullaha, M., Rashid, A., Fasola, M., Hussain, A., Bokhari, H., & Eqani, S. A. M. A. S. (2014). | Cattle egrets as a biosentinels of persistent organic pollutants exposure. |
| 72 | Kioko, J., Boyd, E., Schaeffer, E., Tareen, S., & Kiffner, C. (2016). | Cattle Egret (Bubulcus ibis) interactions with large mammals in the Tarangire-Manyara Ecosystem, Northern Tanzania. |
| 73 | Kopij, G. (2003). | Diet of Cattle Egret Bubulcus ibis chicks in an intensively managed farmland in South Africa. |
| 74 | Kopij, G. (2016). | Mortality in South African Cattle Egrets Bubulcus ibis from 1951 to 1987. |
| 75 | Kopij, G., & Paxton, M. (2018). | Seasonal changes in the diversity and numbers of waterbirds in a tropical river in Southern Africa. |
| 76 | Kopij, G. (2014). | Distribution and numbers of breeding cormorants (Phalacrocoracidae) and herons (Ardeidae) in Lesotho. |
| 77 | Kopij, G. (2017). | Migratory connectivity of south African cattle Egrets (Bubulcus Ibis, Ciconiiformes, Ardeidae). |
| 78 | Kopij, G., & Paxton, M. (2019). | Waterbirds in the panhandle of the Okavango Delta: Dry season counts over two seven-year periods. |
| 79 | Krebs, E., Hunte, W., & Green, D. (2004). | Plume variation, breeding performance and extra-pair copulations in the cattle egret. |
| 80 | Krebs, E. A., Riven-Ramsey, D., & Hunte, W. (1994). | The colonization of Barbados by cattle egrets (Bubulcus ibis) 1956-1990. |
| 81 | Krebs, E. A., Riven-Ramsey, D., & Hunte, W. (1994). | The colonization of Barbados by cattle egrets (Bubulcus ibis) 1956-1990. |
| 82 | Kuang, J., Ye, Y., Lei, Y., Liu, J., Duan, W., Qing, B., ... & Ding, C. (2025). | Why do Cattle Egrets forage with cattle? An analysis from an anti-predation perspective. |
| 83 | Kushlan, J. A. (1979). | Arboreal foraging by Cattle Egrets. |
| 84 | Lesch, V., Kylin, H., & Bouwman, H. (2024b). | The Cattle Egret Bubulcus ibis as a near-global indicator of terrestrial pollution. |
| 85 | Lesch, V., Pieters, R., & Bouwman, H. (2024a). | Dioxins, PFOS, and 20 other Persistent Organic Pollutants in Eggs of Nine Wild Bird Species from the Vaal River, South Africa. |
| 86 | Liversidge, R. (1958). | The bird population of the dams on the Free State goldfields. |
| 87 | Lombardini, K., Bennetts, R. E., & Tourenq, C. (2001). | Foraging success and foraging habitat use by Cattle Egrets and Little Egrets in the Camargue, France. |
| 88 | Lowe-McConnell, R. H. (1967). | Biology of the immigrant Cattle Egret Ardeola ibis in Guyana, South America. |
| 89 | Maddock, M., & Geering, D. (1994). | Range expansion and migration of the Cattle Egret. |
| 90 | Massa, C., Doyle, M., & Callicó Fortunato, R. (2014). | On how Cattle Egret (Bubulcus ibis) spread to the Americas: meteorological tools to assess probable colonization trajectories. |
| 91 | McCrimmon Jr, D. A., Fryska, S. T., Ogden, J. C., & Butcher, G. S. (1997). | Nonlinear population dynamics of six species of Florida Ciconiiformes assessed by Christmas Bird Counts. |
| 92 | McKilligan, N. (2001). | Population dynamics of the Cattle Egret (Ardea ibis) in south-east Queensland: a 20-year study. |
| 93 | McKilligan, N. G. (1990). | Promiscuity in the cattle egret (Bubulcus ibis). |
| 94 | McKilligan, N. G., Reimer, D. S., Seton, D. H. C., Davidson, D. H. C., & Willows, J. T. (1993). | Survival and seasonal movements of the cattle egret in eastern Australia. |
| 95 | McKilligan, N. G. (1985). | The breeding success of the Indian Cattle Egret Ardeola ibis in eastern Australia. |
| 96 | McKilligan, N. G. (1987). | Causes of nesting losses in the cattle egret Ardeola ibis in eastern Australia with special reference to the pathogenicity of the tick Argas (Persicargas) robertsi to nestlings. |
| 97 | Mehta, K., Koli, V. K., Kittur, S., & Sundar, K. S. G. (2024). | Can you nest where you roost? Waterbirds use different sites but similar cues to locate roosting and breeding sites in a small Indian city. |
| 98 | Menon, G. K. (1981). | Cattle Egrets Feeding in Association with Human Workers. |
| 99 | Metallaoui, S., Dziri, H., Bousseheba, A., Heddam, S., & Chenchouni, H. (2020). | Breeding ecology of the Cattle Egret (Bubulcus ibis) in Guerbes-Sanhadja wetlands of Algeria. |
| 100 | Metz, K. J., Prior, K. A., & Mallory, M. L. (1991). | Do cattle egrets gain information from conspecifics when foraging?. |
| 101 | Miño, C. I., Geronimo, C. T., Castillo, C. C., Bonatelli, I. A., Valdes, T. A., Laroca, B. A., & Del Lama, S. N. (2022). | Genetic insights into the range expansion of the cattle egret (Pelecaniformes: Ardeidae) in Brazil and population differentiation between the native and colonized areas. |
| 102 | Mock, D. W., & Lamey, T. C. (1991). | The role of brood size in regulating egret sibling aggression. |
| 103 | Mohammedi, A., & Ababou, A. (2020). | Temporal variation of Bubulcus ibis L. diet in a semi-arid Mediterranean region in north Algeria. |
| 104 | Mora, M. A. (1997). | Feeding flights of cattle egrets nesting in an agricultural ecosystem. |
| 105 | Mora, M. A. (1992). | Habitat use by foraging cattle egrets in the Mexicali Valley, Baja California. |
| 106 | Mora, M. A. (1991). | Organochlorines and breeding success in cattle egrets from the Mexicali Valley, Baja California, Mexico. |
| 107 | Moralez-Silva, E., & Del Lama, S. N. (2017). | Multiple Females Laying in Cattle Egret (Bubulcus ibis) Clutches: Behavioral and Genetic Evidence. |
| 108 | Mukherjee, A., & Borad, C. K. (2001). | Effects of waterbirds on water quality. |
| 109 | Møller, A. P., Arriero, E., Lobato, E., & Merino, S. (2009). | A meta-analysis of parasite virulence in nestling birds. |
| 110 | Nefla, A., Tlili, W., Ouni, R., & Nouira, S. (2015). | Habitat use and breeding biology of Herons in the Ramsar wetlands of Northern Tunisia. |
| 111 | Oschadleus, H. D., Paijmans, D., & Rose, S. (2019). | How far and how old: Longevity and displacement records from the South African Bird Ringing Scheme for Ardeidae, Threskiornithidae and Ciconiidae. |
| 112 | Padhiary, M., Hoque, A., Prasad, G., Kumar, K., & Sahu, B. (2025). | Precision agriculture and AI-driven resource optimization for sustainable land and resource management. |
| 113 | Panda, B. P., Parida, S. P., Pradhan, A., Mohanta, Y. K., Parthipan, P., Narayan, M., & Sarma, H. (2025). | Identifying ecological risk zones using spatial prioritization of heavy metal pollution and bioaccumulation in birds. |
| 114 | Papakostas, G., Kazantzidis, S., Goutner, V., & Charalambidou, I. (2005). | Factors affecting the foraging behavior of the Squacco Heron. |
| 115 | Parfitt, Bruce D. (1976). | Cattle Egrets in Central Coahuila. |
| 116 | Park, S. R., Kim, K. Y., Chung, H., Choi, Y. S., & Sung, H. C. (2011). | Vertical nest stratification and breeding success in a six mixed-species heronry in Taeseong, Chungbuk, Korea. |
| 117 | Parkes, M. L., Mora, M. A., & Feagin, R. A. (2012). | Using scale, cover type and GIS to evaluate nuisance Cattle Egret colony site selection. |
| 118 | Parsons, K. C. (2003). | Reproductive success of wading birds using Phragmites marsh and upland nesting habitats. |
| 119 | Platt, S. G., Lin, N., & Rainwater, T. R. (2021). | Arboreal foraging by Cattle Egrets (Bubulcus ibis) on swarming insects in Myanmar. |
| 120 | Ploger, B. J., & Mock, D. W. (1986). | Role of sibling aggression in food distribution to nestling cattle egrets (Bubulcus ibis). |
| 121 | Poiani, A. (2006). | Effects of floods on distribution and reproduction of aquatic birds. |
| 122 | Porio, E. (2011). | Vulnerability, adaptation, and resilience to floods and climate change-related risks among marginal, riverine communities in Metro Manila. |
| 123 | Prosser, D. J., Sullivan, J. D., Gilbert, C. J., Brinker, D. F., McGowan, P. C., Callahan, C. R., Hutzell, B., & Smith, L. E. (2022). | A comparison of direct & indirect survey methods for estimating colonial nesting waterbird populations. |
| 124 | Putnam, L. S. (1978). | Cattle Egret Nesting in Western Lake Erie. |
| 125 | Ranglack, G. S., Angus, R. A., & Marion, K. R. (1991). | Physical and temporal factors influencing breeding success of Cattle Egrets (Bubulcus ibis) in a West Alabama colony. |
| 126 | Rashed, M. N. (2015). | Biomonitoring and bioindicators for environmental pollution with heavy metals. |
| 127 | Rashed, M. N. (2015). | Biomonitoring and bioindicators for environmental pollution with heavy metals. |
| 128 | Ray, C., Telfair, I. I., McCrimmon Jr, D. A., & Fryska, S. T. (2000). | Population dynamics of the Cattle Egret in Texas, 1954-1999. |
| 129 | Richardson, A. J., & Taylor, I. R. (2003). | Are rice fields in southeastern Australia an adequate substitute for natural wetlands as foraging areas for egrets? |
| 130 | Richardson, A. J., Taylor, I. R., & Growns, J. E. (2001). | The Foraging Ecology of Egrets in Rice Fields in Southern New South Wales, Australia. |
| 131 | Samraoui, F., Menaï, R., & Samraoui, B. (2007). | Reproductive ecology of the Cattle Egret (Bubulcus ibis) at Sidi Achour, north-eastern Algeria. |
| 132 | Samraoui, F., Nedjah, R., Boucheker, A., El‐Serehy, H. A., & Samraoui, B. (2025). | Sky High or Grounded: Nest Site Selection of Herons and Egrets in a Mixed‐Species Colony. |
| 133 | Sbiki, M., Chenchouni, H., & Si Bachir, A. (2015). | Population increase and nest-site selection of Cattle Egrets Bubulcus ibis at a new colony in drylands of north-east Algeria. |
| 134 | Scherer, J. D. F. M., Scherer, A. L., & Petry, M. V. (2014). | Vertical nest stratification of four heron species in southern Brazil. |
| 135 | Scott, D. (1984). | The feeding success of cattle egrets in flocks. |
| 136 | Seedikkoya, K., & Azeez, P. A. (2009). | Cattle Egrets and dry fish business in coastal areas. |
| 137 | Shahbaz, M., Hashmi, M. Z., Malik, R. N., & Yasmin, A. (2013). | Relationship between heavy metals concentrations in egret species, their environment and food chain differences from two Headworks of Pakistan. |
| 138 | Shimada, T., Bowman, A., & Ishida, M. (2000). | Effects of flooding on a wetland bird community. |
| 139 | Si Bachir, A., Barbraud, C., Cereghino, R., & Santoul, F. (2012). | Cattle Egrets (Ardea ibis) use human-made habitat in a newly colonised area in northern Algeria. |
| 140 | Siegfried, W. R. (1972a). | Aspects of the feeding ecology of Cattle Egrets (Ardeola ibis) in South Africa. |
| 141 | Siegfried, W. R. (1972b). | Breeding success and reproductive output of the Cattle Egret. |
| 142 | Siegfried, W. R. (1971a). | Communal Roosting of the Cattle Egret. |
| 143 | Siegfried, W. R. (1968). | Ecological Composition of the Avifaunal Community in a Stellenbosch Suburb. |
| 144 | Siegfried, W. R. (1969). | Energy Metabolism of the Cattle Egret. |
| 145 | Siegfried, W. R. (1970). | Mortality And Dispersal of Ringed Cattle Egrets. |
| 146 | Siegfried, W. R. (1971b). | Population dynamics of the Cattle Egret. |
| 147 | Siegfried, W. R. (1971c). | The Food of the Cattle Egret. |
| 148 | Siegfried, W. R. (1965). | The Status of the Cattle Egret in the Cape Province. |
| 149 | Skead, C. J. (1966). | A study of the cattle egret, Ardeola ibis, Linnaeus. |
| 150 | Skead, C. J. (1952). | The Status of the Cattle Egret, Ardeola Ibis in the Eastern Cape Province. |
| 151 | Smallwood, J. A., Woodrey, M., Smallwood, N. J., & Kettler, M. A. (1982). | Foraging by Cattle Egrets and American Kestrels at a fire's edge. |
| 152 | Stafford, J. D., Kaminski, R. M., & Reinecke, K. J. (2010). | Avian foods, foraging and habitat conservation in world rice fields. |
| 153 | Talbi, R., Gavish, Y., Izhaki, I., & Bar-Massada, A. (2023). | When a pest-control species becomes a pest: a shift in the foraging habitat of cattle egret (Bubulcus ibis) and the threat to grazed natural ecosystems. |
| 154 | Telesford-Checkley, J. M., Mora, M. A., Boellstorff, D. E., & Provin, T. L. (2016). | An evaluation of the contribution of macro- and micro-elements from colonial nesting waterbirds to surface water. |
| 155 | Telesford-Checkley, J. M., Mora, M. A., Grant, W. E., Boellstorff, D. E., & Provin, T. L. (2017a). | Estimating the contribution of nitrogen and phosphorus to waterbodies by colonial nesting waterbirds. |
| 156 | Telesford‐Checkley, J. M., Mora, M. A., Gentry, T. J., McDonald, T. J., & Boellstorff, D. E. (2017). | Impacts of heronries on water quality as evaluated through Escherichia coli and fecal sterol analyses. |
| 157 | Telfair, R. C., II. (1981). | Cattle Egrets, Inland Heronries, and the Availability of Crayfish. |
| 158 | Telfair II, R. C., & Bister, T. J. (2004). | Long-term breeding success of the cattle egret in Texas. |
| 159 | Thompson, C. F., Lanyon, S. M., & Thompson, K. M. (1982). | The influence of foraging benefits on association of cattle egrets (Bubulcus ibis) with cattle. |
| 160 | Toral, G. M., Aragones, D., Bustamante, J., & Figuerola, J. (2011). | Using Landsat images to map habitat availability for waterbirds in rice fields. |
| 161 | Torres, R., & Mongeaud, A. (2006). | Factors affecting the nesting success of the Cattle Egret (Bubulcus ibis) in Laguna Mar Chiquita, central Argentina. |
| 162 | Underhill, L. G., Oatley, T. B., & Harrison, J. A. (1991). | The role of large-scale data collection projects in the study of southern African birds. |
| 163 | Uzun, A., Uzun, B., & Kopij, G. (2008). | Year-to-year variation in clutch size and breeding success of the Little Egret, Egretta garzetta Linnaeus, 1766, in Turkey. |
| 164 | Villar Couto, C. M., Cumming, G. S., Lacorte, G. A., Congrains, C., Izbicki, R., Braga, E. M., et al. (2019). | Avian haemosporidians in the cattle egret (Bubulcus ibis) from central-western and southern Africa: High diversity and prevalence. |
| 165 | Wahungu, G. M., Mumia, E. N., & Manoa, D. (2003). | The effects of flock size, habitat type and cattle herd sizes on feeding and vigilance in cattle egrets (Ardeola ibis). |
| 166 | Weber, W. J. (1975). | Notes on Cattle Egret Breeding. |
| 167 | Wheeler, J. R. (1962). | Observations on the cattle egret in eastern Australia. |
| 168 | Woolfenden, G. E., White, S. C., Mumme, R. L., & Robertson, W. B., Jr. (1976). | Aggression among starving cattle egrets. |
| 169 | Yasmeen, R., Muhammad, H. A., Bokhari, S. S., Rafi, U., Shakoor, A., & Qurashi, A. W. (2019). | Assessment of heavy metals in different organs of cattle egrets (Bubulcus ibis) from a rural and urban environment in Pakistan. |
| 170 | Youcefi, A., Baaloudj, A., Zebsa, R., Mahdjoub, H., Bensakhri, Z., Bensouilah, S., ... & Khelifa, R. (2019). | Weather conditions affect the collective roosting behaviour of the Cattle Egret Bubulcus ibis. |
| 171 | Zaman, M. H., Mustafa, G., Sheikh, M. A., Qadir, A., Shahid, S. U., & Abbasi, N. A. (2022). | A multi-tissue biomonitoring investigation of toxic trace elements and their trophic transfer potential in a semi aquatic bird species, the Cattle Egret (Bubulcus ibis). |
| 172 | Zamani-Ahmadmahmoodi, R., Esmaili-Sari, A., Savabieasfahani, M., & Bahramifar, N. (2010). | Cattle egret (Bubulcus ibis) and little egret (Egretta garzetta) as monitors of mercury contamination in Shadegan Wetlands of south-western Iran. |
| 173 | Zimmerman, Dale A. (1973). | Cattle Egrets in Norther Mexico. |

**S4. Master Study Metadata Table**

| **Doc ID** | **Affiliation Country** | **Institution(s)** | **Year** | **Study Country/Region** | **Study Region** | **Study Type** |
| --- | --- | --- | --- | --- | --- | --- |
| 1 | Pakistan | University of the Punjab; CIMR; WWF Pakistan | 2022 | Pakistan | South Asia | Threats, Contaminants & Ecosystem Services |
| 2 | South Africa | University of Cape Town | 1966 | South Africa | Eastern Cape Province | Breeding Ecology and Behaviour |
| 3 | United States | Rutgers University; Rutgers Medical School | 1989 | Kenya | East Africa | Habitat Use and Foraging Ecology |
| 4 | United States | University of South Florida; U.S. National Park Service | 1976 | United States | North America | Habitat Use and Foraging Ecology; Behavioural ecology |
| 5 | Myanmar; United States | WCS Myanmar; Clemson University | 2021 | Myanmar | Southeast Asia | Habitat Use and Foraging Ecology |
| 6 | United States | U.S. National Park Service | 1979 | United States | North America | Habitat Use and Foraging Ecology |
| 7 | Australia | Charles Sturt University; Murray-Darling Freshwater Research Centre | 2003 | Australia | Oceania | Breeding Ecology and Behaviour |
| 8 | South Africa | University of Cape Town - Percy FitzPatrick Institute | 1972 | South Africa | Southern Africa | Habitat Use and Foraging Ecology |
| 9 | Pakistan | Lahore Garrison University; Environment Protection Agency Punjab | 2019 | Pakistan | South Asia | Threats, Contaminants & Ecosystem Services |
| 10 | Brazil; South Africa; Nigeria; Guinea-Bissau; Senegal | UFSCar; University of Cape Town; IFMG; UFMG; A.P. Leventis ORI; IBAP; AfriWet Consultants | 2019 | Multi-country | Africa | Threats, Contaminants & Ecosystem Services |
| 11 | South Africa | University of KwaZulu-Natal | 2012 | South Africa | Southern Africa | Breeding Ecology and Behaviour |
| 12 | Guyana | Botanic Gardens, Georgetown | 1965 | Guyana | Caribbean | Breeding Ecology and Behaviour |
| 13 | Algeria | University of 20th August 1955 Skikda; University of Tebessa; University of Oum-El-Bouaghi | 2019 | Algeria | North Africa | Breeding Ecology and Behaviour |
| 14 | South Africa | University of Cape Town | 1971 | South Africa | Southern Africa | Habitat Use and Foraging Ecology |
| 15 | Iran; Australia | Islamic Azad University; University of New England | 2013 | Iran | Western Asia | Breeding Ecology and Behaviour |
| 16 | India | Mohanlal Sukhadia University | 2024 | India | South Asia | Habitat Use and Foraging Ecology; Urban ecology |
| 17 | United Kingdom; South Africa; Australia | University of Oxford; University of Cape Town; Independent | 2008 | South Africa | Southern Africa | Habitat Use and Foraging Ecology |
| 18 | Tanzania; United States | School for Field Studies; North Dakota State University; Vassar College | 2016 | Tanzania | Africa | Habitat Use and Foraging Ecology |
| 19 | United States | Ohio State University | 1978 | Canada | North America | Breeding Ecology and Behaviour |
| 20 | United States | Clarion University; Hendrix College | 1989 | United States | North America | Breeding Ecology and Behaviour |
| 21 | India | MES College; SACON | 2009 | India | South Asia | Habitat Use and Foraging Ecology |
| 22 | Algeria; France | Universite de Batna; CNRS CEBC; Universite de Toulouse | 2012 | Algeria | North Africa | Habitat Use and Foraging Ecology |
| 23 | United States | Bird-Banding; Magnolia Gardens SC | 1965 | United States | North America | Population Dynamics and Distribution |
| 24 | India | M. S. University, Baroda | 1981 | India | South Asia | Habitat Use and Foraging Ecology |
| 25 | United States | University of Alaska | 1976 | Mexico | North America | Population Dynamics and Distribution |
| 26 | United States | Western New Mexico University | 1973 | Mexico | North America | Population Dynamics and Distribution |
| 27 | United States | Texas A&M University | 1981 | United States | North America | Breeding Ecology and Behaviour |
| 28 | United States | Rutgers University | 1978 | United States | North America | Habitat Use and Foraging Ecology |
| 29 | United States | Dartmouth College; University of Colorado; University of New Mexico | 2003 | United States | North America | Habitat Use and Foraging Ecology |
| 30 | Poland | Agricultural University of Wroclaw | 2003 | South Africa | Southern Africa | Habitat Use and Foraging Ecology |
| 31 | Argentina | Instituto Nacional de Limnologia; Universidad Nacional de La Plata | 2008 | Argentina | South America | Habitat Use and Foraging Ecology |
| 32 | Algeria; France | Universite de Bejaia; Institut National Agronomique; MNHN Paris | 2012 | Algeria | North Africa | Habitat Use and Foraging Ecology |
| 33 | France; United States | Station Biologique Tour du Valat; National Park Service USA | 2006 | France | Western Europe | Breeding Ecology and Behaviour |
| 34 | Canada | Carleton University | 1991 | Costa Rica | Central America | Habitat Use and Foraging Ecology |
| 35 | Australia | Macquarie University; University of Queensland; Deakin University | 1998 | Australia | Oceania | Breeding Ecology and Behaviour |
| 36 | South Africa | University of Cape Town | 1968 | South Africa | Southern Africa | Population Dynamics and Distribution |
| 37 | South Africa | University of Cape Town | 1969 | South Africa | Southern Africa | Habitat Use and Foraging Ecology; Breeding Ecology and Behaviour |
| 38 | South Africa; Japan; Ethiopia | North-West University; Hokkaido University; University of Gondar; University of the Witwatersrand | 2019 | South Africa | Southern Africa | Threats, Contaminants & Ecosystem Services |
| 39 | United States | University of California Davis; National Biological Service; Texas A&M University | 1997 | Mexico | North America | Habitat Use and Foraging Ecology |
| 40 | Cyprus | University of Nicosia; Near East University | 2013 | Cyprus | Middle East | Breeding Ecology and Behaviour |
| 41 | Tanzania; South Korea | University of Dar es Salaam; Seoul National University | 2012 | Tanzania | East Africa | Habitat Use and Foraging Ecology |
| 42 | United States | University of Georgia; USDA-APHIS Wildlife Services | 2000 | U.S. Virgin Islands | Caribbean | Habitat Use and Foraging Ecology |
| 43 | United States | Miami University | 1982 | United States | North America | Habitat Use and Foraging Ecology |
| 44 | France | Station Biologique de la Tour du Valat | 2001 | France | Western Europe | Habitat Use and Foraging Ecology |
| 45 | United States | University of Tampa | 1973 | United States | North America | Habitat Use and Foraging Ecology |
| 46 | United States | University of Michigan; Michigan State University; Cornell University | 1974 | United States | North America | Habitat Use and Foraging Ecology |
| 47 | Pakistan | Islamia University of Bahawalpur; University of Agriculture Faisalabad; COMSATS | 2017 | Pakistan | South Asia | Breeding Ecology and Behaviour |
| 48 | United States | (None listed) | 1992 | Mexico | North America | Habitat Use and Foraging Ecology |
| 49 | Algeria | Universite de Bejaia; University Batna 2; University of Ghardaia; ENSA Alger | 2022 | Algeria | North Africa | Breeding Ecology and Behaviour |
| 50 | South Africa | University of Cape Town; University of KwaZulu-Natal | 2019 | South Africa | Southern Africa | Population Dynamics and Distribution |
| 51 | Poland; Namibia | Wroclaw University of Environmental & Life Sciences; University of Namibia | 2016 | South Africa | Southern Africa | Population Dynamics and Distribution |
| 52 | United States | Texas Parks and Wildlife Department | 2003 | United States | North America | Breeding Ecology and Behaviour |
| 53 | United States | Rutgers University; UMDNJ-Robert Wood Johnson Medical School | 1993 | Kenya | East Africa | Habitat Use and Foraging Ecology |
| 54 | South Africa | Percy FitzPatrick Institute, University of Cape Town | 1970 | South Africa | Southern Africa | Population Dynamics and Distribution |
| 55 | United States | University of Miami; University of Florida | 1973 | United States | North America | Population Dynamics and Distribution |
| 56 | Indonesia | IPB University (Bogor Agricultural University) | 2020 | Indonesia | Southeast Asia | Threats, Contaminants & Ecosystem Services |
| 57 | Brazil | Federal University of Sao Carlos | 2017 | Brazil | South America | Breeding Ecology and Behaviour |
| 58 | Ghana | University of Ghana | 2013 | Ghana | West Africa | Habitat Use and Foraging Ecology |
| 59 | United States | Rutgers University; CMDNJ-Rutgers Medical School | 1984 | South Africa | Southern Africa | Habitat Use and Foraging Ecology |
| 60 | South Africa; Norway | North-West University; Norwegian School of Veterinary Science; National Veterinary Institute | 2007 | South Africa | Southern Africa | Threats, Contaminants & Ecosystem Services |
| 61 | United States | Leesburg, Florida (independent researcher) | 1974 | United States | North America | Breeding Ecology and Behaviour |
| 62 | Australia | Belmont, Victoria (independent) | 1962 | Australia | Oceania | Habitat Use and Foraging Ecology |
| 63 | Australia | Tenterfield, NSW (independent) | 1965 | Australia | Oceania | Breeding Ecology and Behaviour |
| 64 | India | Aligarh Muslim University; World Pheasant Association; Dept. Wildlife Protection J&K | 2003 | India | South Asia | Breeding Ecology and Behaviour |
| 65 | Algeria; France | Universite de Batna; CNRS CEBC; INA El Harrach; Tour du Valat | 2008 | Algeria | North Africa | Breeding Ecology and Behaviour |
| 66 | Argentina | Universidad de Buenos Aires - IEGEBA; CIMA CONICET-UBA | 2014 | Argentina | Multi-region study | Population Dynamics and Distribution |
| 67 | United States | Rutgers University | 1980 | South Africa | Southern Africa | Breeding Ecology and Behaviour |
| 68 | Sierra Leone | West African Rice Research Station, Rokupr | 1965 | Sierra Leone | West Africa | Habitat Use and Foraging Ecology |
| 69 | Australia | University of Southern Queensland | 2000 | Australia | Oceania | Population Dynamics and Distribution |
| 70 | United States | Texas Parks and Wildlife Department; Lake Superior State University; Allied Signal Inc. | 2000 | United States | North America | Population Dynamics and Distribution |
| 71 | Canada; Barbados; Australia | McGill University; Simon Fraser University; Bellairs Research Institute | 2004 | Barbados | Caribbean | Breeding Ecology and Behaviour |
| 72 | Australia | Darling Downs Institute of Advanced Education | 1990 | Australia | Oceania | Breeding Ecology and Behaviour |
| 73 | Australia | Shortland Wetlands Centre, NSW | 1994 | Australia | Oceania | Population Dynamics and Distribution |
| 74 | United States | U.S. Forest Service; Institute of Tropical Forestry | 1988 | Puerto Rico | Caribbean | Population Dynamics and Distribution |
| 75 | United States | University of Montana | 1973 | United States | North America | Habitat Use and Foraging Ecology |
| 76 | Algeria | University of Annaba; University of Guelma | 2007 | Algeria | North Africa | Breeding Ecology and Behaviour |
| 77 | China; Madagascar | China University of Geosciences (Wuhan); Independent Ecology Consultant | 2016 | Madagascar | Southern Africa | Habitat Use and Foraging Ecology |
| 78 | United States | University of Georgia - SCWDS | 1993 | Caribbean (multi-island) | North America | Threats, Contaminants & Ecosystem Services |
| 79 | Russia; South Africa | St. Petersburg State University; University of the Free State | 2003 | South Africa | Southern Africa | Threats, Contaminants & Ecosystem Services |
| 80 | Algeria | University of Tebessa; El Hadj Lakhdar University Batna | 2015 | Algeria | North Africa | Population Dynamics and Distribution |
| 81 | South Africa | Percy FitzPatrick Institute of African Ornithology | 1971 | South Africa | Southern Africa | Population Dynamics and Distribution |
| 82 | United States | University of Oklahoma | 1986 | United States | North America | Breeding Ecology and Behaviour |
| 83 | United States | University of Oklahoma | 1991 | United States | North America | Breeding Ecology and Behaviour |
| 84 | United States | Illinois State University; Louisiana State University | 1982 | United States | North America | Habitat Use and Foraging Ecology |
| 85 | Australia | University of Queensland; Macquarie University | 1994 | Australia | Oceania | Habitat Use and Foraging Ecology |
| 86 | South Africa | Cape Department of Nature Conservation, Stellenbosch | 1964 | South Africa | Southern Africa | Population Dynamics and Distribution |
| 87 | Australia | Charles Sturt University; Murray-Darling Freshwater Research Centre | 2001 | Australia | Oceania | Habitat Use and Foraging Ecology |
| 88 | South Africa | Albany Museum, Grahamstown | 1952 | South Africa | Southern Africa | Population Dynamics and Distribution |
| 89 | Australia | Darling Downs Institute of Advanced Education | 1985 | Australia | Oceania | Breeding Ecology and Behaviour |
| 90 | South Africa; Sweden | North-West University; Linkoping University | 2023 | Multi-country | Multi-region study | Threats, Contaminants & Ecosystem Services |
| 91 | Canada; Barbados; Australia | McGill University; Bellairs Research Institute; University of the West Indies; Australian National University | 1994 | Barbados | Caribbean | Population Dynamics and Distribution |
| 92 | France | Station Biologique de la Tour du Valat | 1992 | France | Western Europe | Population Dynamics and Distribution |
| 93 | Kenya | Moi University | 2003 | Kenya | East Africa | Habitat Use and Foraging Ecology |
| 94 | United States | University of Oklahoma | 1984 | United States | North America | Habitat Use and Foraging Ecology |
| 95 | United States; South Africa | University of Minnesota; University of Cape Town | 1971 | South Africa | Southern Africa | Habitat Use and Foraging Ecology |
| 96 | Algeria | University Hassiba Benbouali of Chlef | 2020 | Algeria | North Africa | Habitat Use and Foraging Ecology |
| 97 | United States | Florida Game and Fresh Water Fish Commission | 1972 | United States | North America | Habitat Use and Foraging Ecology |
| 98 | Poland; Namibia | Wroclaw University of Environmental & Life Sciences; Shamvura Camp | 2018 | Namibia | Southern Africa | Population Dynamics and Distribution; Habitat Use and Foraging Ecology |
| 99 | Puerto Rico | University of Puerto Rico | 1964 | Puerto Rico | Caribbean | Habitat Use and Foraging Ecology |
| 100 | United Kingdom | MAFF Pest Infestation Control Laboratory | 1975 | Seychelles | Indian Ocean | Habitat Use and Foraging Ecology |
| 101 | Australia | University of Southern Queensland | 1993 | Australia | Oceania | Population Dynamics and Distribution |
| 102 | United States | Texas A&M University; Great Ecology & Environments | 2012 | United States | North America | Habitat Use and Foraging Ecology |
| 103 | Algeria; Canada; Switzerland | Setif-1 University; University of Batna 2; University of British Columbia; University of Zurich | 2019 | Algeria | North Africa | Breeding Ecology and Behaviour |
| 104 | Ghana; Nigeria | University of Cape Coast; University of Lagos; TETFUND | 2024 | Ghana | West Africa | Habitat Use and Foraging Ecology |
| 105 | South Africa | Rhodes University | 2021 | South Africa | Southern Africa | Habitat Use and Foraging Ecology |
| 106 | South Africa | University of KwaZulu-Natal | 2025 | South Africa | Southern Africa | Habitat Use and Foraging Ecology; Urban ecology |
| 107 | Israel | University of Haifa | 2023 | Israel | Middle East | Habitat Use and Foraging Ecology |
| 108 | China | Beijing Forestry University; Shaanxi Hanzhong Crested Ibis NNR | 2025 | China | East Asia | Habitat Use and Foraging Ecology |
| 109 | Zimbabwe; France; South Africa | Cirad; University of Pretoria | 2014 | Zimbabwe | Southern Africa | Threats, Contaminants & Ecosystem Services |
| 110 | India | National Tree Growers Cooperative Federation - Wetland Conservation Project | 2001 | India | South Asia | Threats, Contaminants & Ecosystem Services |
| 111 | Mexico | Universidad Nacional Autonoma de Mexico (UNAM) | 2007 | Mexico | North America | Habitat Use and Foraging Ecology |
| 112 | Canada | University of Montreal - Faculty of Veterinary Medicine | 1999 | Global | Global | Breeding Ecology and Behaviour |
| 113 | South Africa | Port Elizabeth Museum | 1958 | South Africa | Southern Africa | Population Dynamics and Distribution |
| 114 | Germany; Egypt | Independent researchers; Georg-August-Universitat Gottingen | 2020 | Egypt | North Africa | Population Dynamics and Distribution |
| 115 | Germany; Netherlands; Egypt | Institute of Avian Research - Vogelwarte Helgoland; Georg-August-Universitat Gottingen | 2020 | Egypt | North Africa | Breeding Ecology and Behaviour |
| 116 | Japan | Izunuma-Uchinuma Environmental Foundation; Japanese Association for Wild Geese Protection | 2000 | Japan | East Asia | Threats, Contaminants & Ecosystem Services |
| 117 | Australia | UNSW Sydney | 2021 | Botswana | Southern Africa | Population Dynamics and Distribution |
| 118 | South Africa | University of Cape Town; South African Bird Ringing Unit; SABAP | 1991 | Southern Africa | Southern Africa | Population Dynamics and Distribution |
| 119 | United States | Rutgers University; UMDNJ-Robert Wood Johnson Medical School | 1990 | Madagascar | East Africa | Breeding Ecology and Behaviour |
| 120 | Philippines | Ateneo de Manila University | 2011 | Philippines | Southeast Asia | Threats, Contaminants & Ecosystem Services |
| 121 | Ethiopia; United States | Amhara Regional State; Jimma University; TREE Foundation; Sonoma State University; UCSF | 2011 | Ethiopia | East Africa | Habitat Use and Foraging Ecology |
| 122 | United States | Texas A&M University; Texas Commission on Environmental Quality | 2017 | United States | North America | Threats, Contaminants & Ecosystem Services |
| 123 | Namibia | University of Namibia; Shamvura Camp | 2019 | Namibia | Southern Africa | Population Dynamics and Distribution |
| 124 | Namibia | University of Namibia | 2013 | Lesotho | Southern Africa | Population Dynamics and Distribution |
| 125 | Turkey; Poland | Sakarya University; Dokuz Eylul University; Wroclaw University of Environmental & Life Sciences | 2013 | Turkey | Western Asia | Breeding Ecology and Behaviour |
| 126 | France | Station Biologique de la Tour du Valat | 1996 | France | Western Europe | Threats & Conservation; Population Dynamics and Distribution |
| 127 | Germany; France; Luxembourg | Trier University; Conservatoire d'espaces naturels PACA; IMBE; IUCN SSC; MNHN Luxembourg | 2023 | France | Western Europe | Habitat Use and Foraging Ecology |
| 128 | Denmark; Niger; Senegal; Benin | National Environmental Research Institute Denmark; Orbicon; DPV; AGRHYMET; IITA | 2009 | Niger; Senegal | West Africa | Threats, Contaminants & Ecosystem Services |
| 129 | Algeria; Saudi Arabia | University of Guelma; University 8 Mai 1945; University Badji Mokhtar; King Saud University | 2024 | Algeria | North Africa | Breeding Ecology and Behaviour |
| 130 | Italy | Istituto Zooprofilattico Sperimentale delle Venezie; SELC Soc. Coop. | 2023 | Italy | Southern Europe | Threats, Contaminants & Ecosystem Services |
| 131 | Uganda; United Kingdom; Ireland | Islamic University in Uganda; University of Oxford; University College Cork | 2012 | Uganda | East Africa | Population Dynamics and Distribution |
| 132 | Australia | University of New South Wales | 2020 | Botswana | Southern Africa | Habitat Use and Foraging Ecology; Threats & Conservation |
| 133 | India; United States | Assam University; Parala Maharaja Engineering College; UTEP | 2025 | India | South Asia | Threats, Contaminants & Ecosystem Services |
| 134 | United States | North Carolina State University | 1977 | United States | North America | Breeding Ecology and Behaviour |
| 135 | United Kingdom; United States | Cambridge University; The Marine Mammal Center | 1995 | Global (conceptual) | Multi-region study | Habitat Use and Foraging Ecology |
| 136 | Italy | Universita di Pavia; Universita di Milano; Regione Lombardia | 2009 | Italy | Southern Europe | Population Dynamics and Distribution |
| 137 | Poland | Wroclaw University of Environmental & Life Sciences | 2017 | South Africa | Southern Africa | Habitat Use and Foraging Ecology |
| 138 | India; United States | Siksha O Anusandhan University; Centurion University; USTM; SRM; UTEP; Bodoland University | 2025 | India | South Asia | Threats, Contaminants & Ecosystem Services |
| 139 | Botswana; South Africa | Botswana Dept. Wildlife & National Parks; Potchefstroom University | 2000 | Botswana | Southern Africa | Threats, Contaminants & Ecosystem Services |
| 140 | Argentina; Brazil; United States | IBS-UNaM-CONICET; UFSCar; University of Hawaii at Manoa; UNIFESP | 2022 | Multi-country (Africa + Brazil) | Multi-region study | Population Dynamics and Distribution |
| 141 | United States | Texas A&M University; Texas A&M AgriLife Extension Service | 2016 | United States | North America | Threats, Contaminants & Ecosystem Services |
| 142 | United States | Texas A&M University; Texas Commission on Environmental Quality; Texas A&M AgriLife | 2016 | United States | North America | Threats, Contaminants & Ecosystem Services |
| 143 | France | Tour du Valat Biological Research Institute | 2019 | France | Western Europe | Breeding Ecology and Behaviour; Threats & Conservation |
| 144 | Argentina | Universidad Nacional de Cordoba | 2005 | Argentina | South America | Breeding Ecology and Behaviour |
| 145 | United States | U.S. Fish and Wildlife Service; Patuxent Wildlife Research Center | 1978 | United States | North America | Habitat Use and Foraging Ecology |
| 146 | United States | U.S. Fish and Wildlife Service; Patuxent Wildlife Research Center | 1983 | United States | North America | Habitat Use and Foraging Ecology |
| 147 | Spain; Sweden | Estacion Biologica de Donana (CSIC); Kristianstad University | 2014 | Global | Global | Threats, Contaminants & Ecosystem Services |
| 148 | Pakistan | Kinnaird College for Women, Lahore | 2011 | Pakistan | South Asia | Threats, Contaminants & Ecosystem Services |
| 149 | India | Bangalore University | 2010 | India | South Asia | Habitat Use and Foraging Ecology; Population Dynamics and Distribution |
| 150 | United States; Canada | Cornell University; USGS Patuxent; University of Virginia; Brock University | 2001 | United States | North America | Population Dynamics and Distribution |
| 151 | Pakistan; Norway; Italy | Quaid-i-Azam University; NILU; NIFA; Universita di Pavia; COMSATS | 2013 | Pakistan | South Asia | Threats, Contaminants & Ecosystem Services |
| 152 | Pakistan; Italy; China; Saudi Arabia | Quaid-i-Azam University; Universita di Pavia; NARC; COMSATS; CAS; KAU | 2014 | Pakistan | South Asia | Threats, Contaminants & Ecosystem Services |
| 153 | United States | University of Illinois; Mississippi State University; USGS | 2010 | Multi-country | Multi-region study | Habitat Use and Foraging Ecology |
| 154 | Egypt | Aswan University | 2015 | Multi-country | Multi-region study | Threats, Contaminants & Ecosystem Services |
| 155 | Pakistan | Quaid-i-Azam University | 2009 | Pakistan | South Asia | Threats, Contaminants & Ecosystem Services |
| 156 | Iran | University of Agricultural Sciences and Natural Resources, Gorgan | 2017 | Iran | Middle East | Habitat Use and Foraging Ecology |
| 157 | Sierra Leone; Pakistan; Australia | ETU Sierra Leone; NUST; University of Sydney; Akhuwat College University | 2025 | Sierra Leone | West Africa | Threats, Contaminants & Ecosystem Services |
| 158 | South Africa; Nigeria | University of Pretoria; National Veterinary Research Institute Vom | 2014 | Nigeria | West Africa | Threats, Contaminants & Ecosystem Services |
| 159 | United States | Texas A&M University; Texas A&M AgriLife; Texas Commission on Environmental Quality | 2016 | United States | North America | Threats, Contaminants & Ecosystem Services |
| 160 | Brazil; South Africa; Australia; Nigeria; Senegal; Guinea-Bissau; Ghana | UFSCar; UESC; University of Cape Town; James Cook University; A.P. Leventis ORI; AfriWetConsultants; University of Ghana | 2016 | Multi-country (Africa) | Multi-region study | Population Dynamics and Distribution |
| 161 | South Africa | Agricultural Research Council - PPRI; Potchefstroom University | 2000 | South Africa | Southern Africa | Threats, Contaminants & Ecosystem Services |
| 162 | South Africa | North-West University | 2024 | South Africa | Southern Africa | Threats, Contaminants & Ecosystem Services |
| 163 | Ghana | University of Ghana; Ghana Wildlife Society | 2015 | Ghana | West Africa | Threats, Contaminants & Ecosystem Services |
| 164 | Algeria; Saudi Arabia | University of Guelma; University 8 Mai 1945; University Badji Mokhtar; King Saud University | 2024 | Algeria | North Africa | Breeding Ecology and Behaviour |
| 165 | Algeria; France | Universite de Batna; CNRS CEBC; INA El Harrach; Tour du Valat | 2008 | Algeria | North Africa | Breeding Ecology and Behaviour |
| 166 | United States; South Africa; India | Texas State University; University of Cape Town; University of KwaZulu-Natal; SAEON | 2022 | Multi-country (Africa) | Africa | Population Dynamics and Distribution |
| 167 | United States | Rutgers University; UMDNJ-Robert Wood Johnson Medical School | 1993 | Kenya | East Africa | Habitat Use and Foraging Ecology |
| 168 | Algeria; France | Universite Mouloud Mammeri; INA; MNHN Paris | 2004 | Algeria | North Africa | Habitat Use and Foraging Ecology |
| 169 | Tunisia | University of Sciences of Tunis; Tunisia Wildlife Conservation Society | 2015 | Tunisia | North Africa | Habitat Use and Foraging Ecology |
| 170 | Egypt; Saudi Arabia | Kafrelsheikh University; Sohag University; Mansoura University; Taif University; Animal Health Research Institute | 2021 | Egypt | North Africa | Threats, Contaminants & Ecosystem Services |
| 171 | South Africa; Norway | North-West University; National Metrology Institute of South Africa; Norwegian School of Veterinary Sciences | 2013 | South Africa | Southern Africa | Threats, Contaminants & Ecosystem Services |
| 172 | Spain | Estacion Biologica de Donana (CSIC) | 2011 | Spain | Western Europe | Habitat Use and Foraging Ecology |
| 173 | Puerto Rico | University of Puerto Rico | 1965 | Puerto Rico | Caribbean | Habitat Use and Foraging Ecology |

**S5. Analytical Code Snippets (R Pseudocode)**

library(bibliometrix)

\# Import Web of Science and Scopus data

WOS_df	<- convert2df("Wos.txt")

Scopus_df <- convert2df("Scopus.bib", dbsource = "scopus", format = "bibtex")

\# Merge and deduplicate

combined <- mergeDbSources(WOS_df, Scopus_df, remove.duplicated = TRUE)

library(openxlsx)

write.xlsx(combined, file = "combined_bibliometric_data.xlsx",

rowNames = FALSE, asTable = TRUE)

\# PRISMA 2020 flow diagram

library(DiagrammeR)

grViz("

digraph prisma {

graph [layout = dot, rankdir = TB]

node [shape = box, style = filled, fillcolor = LightBlue, width = 4]

A1 [label = 'Identification\\nWeb of Science (n=257)']

A2 [label = 'Identification\\nScopus (n=332)']

B [label = 'Duplicates removed (n=31)\\nRecords after duplicates (n=558)']

C [label = 'Screening\\nTitle screening excluded (n=190)\\nRemaining (n=368)']

D [label = 'Abstract screening excluded (n=161)\\nRemaining (n=207)']

E [label = 'Eligibility\\nFull-text assessed (n=207)\\nFull-text unavailable (n=34)']

F [label = 'Included\\nStudies included (n=173)']

A1 -> B

A2 -> B

B -> C

C -> D

D -> E

E -> F

}

")

#==========================================================

\# UNIFORM GLOBAL + AFRICA MAPS (AFFILIATION vs STUDY)

#==========================================================

library(dplyr)

library(tidyr)

library(stringr)

library(sf)

library(ggplot2)

library(rnaturalearth)

library(rnaturalearthdata)

library(readxl)

#----------------------------------------------------------

\# 1. Load dataset

#----------------------------------------------------------

setwd("C:/Users/bheki/OneDrive/Pictures/Documents/Bibtex files/Proper")

df <- read_excel("Study Review Papers.xlsx")

#----------------------------------------------------------

\# 2. Clean Affiliation Country List

#----------------------------------------------------------

df <- df %>%

mutate(

Country_List = strsplit(Country, ";")

) %>%

rowwise() %>%

mutate(

Country_List = list(trimws(Country_List))

) %>%

ungroup()

#----------------------------------------------------------

\# 3. Clean Study Country List

#----------------------------------------------------------

df <- df %>%

mutate(

Study_Country_List = strsplit(`Study Country`, ";")

) %>%

rowwise() %>%

mutate(

Study_Country_List = list(trimws(Study_Country_List))

) %>%

ungroup()

#----------------------------------------------------------

\# 4. Load world map + harmonize names

#----------------------------------------------------------

world <- ne_countries(scale = "medium", returnclass = "sf")

name_fix <- c(

"United States of America" = "USA",

"United Republic of Tanzania" = "Tanzania",

"Russian Federation" = "Russia",

"Iran (Islamic Republic of)" = "Iran",

"Korea, Republic of" = "South Korea",

"Gambia" = "The Gambia",

"Netherlands" = "The Netherlands"

)

world <- world %>%

mutate(name = ifelse(name %in% names(name_fix), name_fix[name], name))

#==========================================================

\# PART A — AFFILIATION-BASED MAPS

#==========================================================

#----------------------------------------------------------

\# 5. Count authors per affiliation country

#----------------------------------------------------------

affil_counts <- df %>%

unnest(Country_List) %>%

count(Country_List, name = "n_authors")

#----------------------------------------------------------

\# 6. Join to world map

#----------------------------------------------------------

world_affil <- world %>%

left_join(affil_counts, by = c("name" = "Country_List"))

#----------------------------------------------------------

\# 7. Compute centroids for labels

#----------------------------------------------------------

affil_centroids <- world_affil %>%

filter(!is.na(n_authors)) %>%

st_centroid() %>%

mutate(

lon = st_coordinates(.)[,1],

lat = st_coordinates(.)[,2]

)

#----------------------------------------------------------

\# 8. GLOBAL AFFILIATION MAP (with numbers)

#----------------------------------------------------------

ggplot(world_affil) +

geom_sf(aes(fill = n_authors), color = "grey40", size = 0.1) +

geom_text(

data = affil_centroids,

aes(x = lon, y = lat, label = n_authors),

size = 3, color = "green", fontface = "bold"

) +

scale_fill_viridis_c(option = "plasma", na.value = "lightgrey") +

theme_minimal(base_size = 14) +

labs(

title = "Global Distribution of Author Affiliations.",

subtitle = "Shading reflects countries where authors were institutionally based; values indicate total affiliated authors.",

caption = "Generated by Bhekinkosi Sibanda"

)

#----------------------------------------------------------

\# 9. AFRICA AFFILIATION MAP (with numbers)

#----------------------------------------------------------

africa_affil <- world_affil %>% filter(continent == "Africa")

africa_affil_centroids <- africa_affil %>%

filter(!is.na(n_authors)) %>%

st_centroid() %>%

mutate(

lon = st_coordinates(.)[,1],

lat = st_coordinates(.)[,2]

)

ggplot() +

geom_sf(data = africa_affil, fill = "white", color = "grey80", size = 0.2) +

geom_sf(

data = africa_affil %>% filter(!is.na(n_authors)),

aes(fill = n_authors),

color = "grey20", size = 0.3

) +

geom_text(

data = africa_affil_centroids,

aes(x = lon, y = lat, label = n_authors),

size = 4, color = "green", fontface = "bold"

) +

scale_fill_viridis_c(option = "plasma", na.value = "white") +

theme_minimal(base_size = 14) +

labs(

title = "African Distribution of Author Affiliations.",

subtitle = "Shading reflects countries where authors were institutionally based; values indicate number of affiliated authors.",

caption = "Generated by Bhekinkosi Sibanda"

)

#==========================================================

\# PART B — STUDY-BASED MAPS

#==========================================================

#----------------------------------------------------------

\# 10. Count studies per Study Country

#----------------------------------------------------------

study_counts <- df %>%

unnest(Study_Country_List) %>%

count(Study_Country_List, name = "n_studies")

#----------------------------------------------------------

\# 11. Join to world map

#----------------------------------------------------------

world_study <- world %>%

left_join(study_counts, by = c("name" = "Study_Country_List"))

#----------------------------------------------------------

\# 12. Compute centroids for labels

#----------------------------------------------------------

study_centroids <- world_study %>%

filter(!is.na(n_studies)) %>%

st_centroid() %>%

mutate(

lon = st_coordinates(.)[,1],

lat = st_coordinates(.)[,2]

)

#----------------------------------------------------------

\# 13. GLOBAL STUDY MAP (with numbers)

#----------------------------------------------------------

ggplot(world_study) +

geom_sf(aes(fill = n_studies), color = "grey40", size = 0.1) +

geom_text(

data = study_centroids,

aes(x = lon, y = lat, label = n_studies),

size = 3, color = "green", fontface = "bold"

) +

scale_fill_viridis_c(option = "plasma", na.value = "lightgrey") +

theme_minimal(base_size = 14) +

labs(

title = "Global Distribution of Study Locations.",

subtitle = "Shading reflects countries where studies were conducted; values indicate total study counts.",

caption = "Generated by Bhekinkosi Sibanda"

)

#----------------------------------------------------------

\# 14. AFRICA STUDY MAP (with numbers)

#----------------------------------------------------------

africa_study <- world_study %>% filter(continent == "Africa")

africa_study_centroids <- africa_study %>%

filter(!is.na(n_studies)) %>%

st_centroid() %>%

mutate(

lon = st_coordinates(.)[,1],

lat = st_coordinates(.)[,2]

)

ggplot() +

geom_sf(data = africa_study, fill = "white", color = "grey80", size = 0.2) +

geom_sf(

data = africa_study %>% filter(!is.na(n_studies)),

aes(fill = n_studies),

color = "grey20", size = 0.3

) +

geom_text(

data = africa_study_centroids,

aes(x = lon, y = lat, label = n_studies),

size = 4, color = "green", fontface = "bold"

) +

scale_fill_viridis_c(option = "plasma", na.value = "white") +

theme_minimal(base_size = 14) +

labs(

title = "African Distribution of Study Locations.",

subtitle = "Shading reflects countries where at least one study was conducted.",

caption = "Generated by Bhekinkosi Sibanda."

)

#----------------------------------------------------------

\# 1. Load Packages

#----------------------------------------------------------

library(dplyr)

library(sf)

library(ggplot2)

#----------------------------------------------------------

\# 2. Filter Africa from world_study

#----------------------------------------------------------

africa_study <- world_study %>%

filter(continent == "Africa")

#----------------------------------------------------------

\# 3. Extract Southern African study counts

#----------------------------------------------------------

southern_africa <- c(

"South Africa", "Zimbabwe", "Mozambique", "Botswana",

"Namibia", "Lesotho", "Eswatini", "Zambia", "Angola", "Madagascar"

)

sa_study_counts <- africa_study %>%

filter(name %in% southern_africa) %>%

st_drop_geometry() %>%

select(Country = name, n_studies) %>%

arrange(desc(n_studies))

sa_study_counts	# <-- This prints your numbers for the paragraph

#----------------------------------------------------------

\# 4. Prepare centroids for map labels

#----------------------------------------------------------

africa_study_centroids <- africa_study %>%

filter(!is.na(n_studies)) %>%

st_centroid() %>%

mutate(

lon = st_coordinates(.)[,1],

lat = st_coordinates(.)[,2]

)

#----------------------------------------------------------

\# 5. Africa Study Map (with numbers)

#----------------------------------------------------------

ggplot() +

geom_sf(data = africa_study, fill = "white", color = "grey80", size = 0.2) +

geom_sf(

data = africa_study %>% filter(!is.na(n_studies)),

aes(fill = n_studies),

color = "grey20", size = 0.3

) +

geom_text(

data = africa_study_centroids,

aes(x = lon, y = lat, label = n_studies),

size = 4, color = "green", fontface = "bold"

) +

scale_fill_viridis_c(option = "plasma", na.value = "white") +

theme_minimal(base_size = 14) +

labs(

title = "African Distribution of Study Locations",

subtitle = "Shading reflects countries where at least one study was conducted",

caption = "Generated by Bhekinkosi Sibanda."

)

################################################################################

\# SECTION 1: Load Packages

################################################################################

library(dplyr)

library(stringr)

library(ggplot2)

################################################################################

\# SECTION 2: Inspect Data Structure

\# Purpose: Confirm column names and ensure the correct field is used for

\#	thematic classification.

################################################################################

names(df)

################################################################################

\# SECTION 3: Define Thematic Dictionary

\# Purpose: Map keywords to analytical pillars for automated classification.

################################################################################

theme_dict <- list(

"Habitat & Foraging Ecology" = c(

"foraging", "habitat", "feeding", "patch", "landfill", "agro",

"urban–waste", "insect availability", "cattle–egret association"

),

"Population Status & Distribution" = c(

"population", "distribution", "range", "status", "abundance", "density"

),

"Breeding Ecology" = c(

"breeding", "nesting", "reproductive", "mating", "brood", "extra‑pair"

),

"Movement & Behavioural Ecology" = c(

"movement", "roosting", "behaviour", "behavior", "seasonal dynamics",

"communal roosting", "marking study"

),

"Conservation challenges, ecosystem services, and management implications" = c(

"threat", "contaminant", "pollution", "ecosystem service", "risk"

)

)

################################################################################

\# SECTION 4: Classification Function

\# Purpose: Assign each study to a thematic pillar based on keyword detection.

################################################################################

classify_theme <- function(text) {

text_lower <- tolower(text)

for (theme in names(theme_dict)) {

keywords <- theme_dict[[theme]]

if (any(str_detect(text_lower, tolower(keywords)))) {

return(theme)

}

}

return("Other / Unclassified")

}

################################################################################

\# SECTION 5: Apply Classification to Dataset

################################################################################

df$Theme_Group <- sapply(df$`Study Type`, classify_theme)

################################################################################

\# SECTION 6: Merge Breeding + Movement Themes

\# Purpose: Align with analytical pillar "Breeding ecology and behaviour".

################################################################################

df <- df %>%

mutate(

Theme_Group = case_when(

Theme_Group %in% c("Breeding Ecology",

"Movement & Behavioural Ecology") ~

"Breeding ecology and behaviour",

TRUE ~ Theme_Group

)

)

################################################################################

\# SECTION 7: Summarise Thematic Counts

################################################################################

theme_counts <- df %>%

count(Theme_Group, name = "n_studies") %>%

mutate(

total = sum(n_studies),

perc = round(100 * n_studies / total, 1)

)

theme_counts

################################################################################

\# SECTION 8: Prepare Data for Plotting

################################################################################

theme_df <- theme_counts %>%

rename(Final_Theme = Theme_Group) %>%

mutate(

Final_Theme = factor(

Final_Theme,

levels = c(

"Habitat & Foraging Ecology",

"Population Status & Distribution",

"Breeding ecology and behaviour",

"Conservation challenges, ecosystem services, and management implications"

),

labels = c(

"Habitat\n& Foraging\nEcology",

"Population\nStatus &\nDistribution",

"Breeding\necology and\nbehaviour",

"Conservation\nchallenges,\necosystem\nservices &\nmanagement\nimplications"

)

)

)

################################################################################

\# SECTION 9: Plot Thematic Distribution

\# Purpose: Produce a clean, publication-ready bar chart.

################################################################################

ggplot(theme_df, aes(x = Final_Theme, y = perc, fill = Final_Theme)) +

geom_col(width = 0.7, colour = "black") +

geom_text(

aes(label = paste0(perc, "% (n=", n_studies, ")")),

vjust = -0.4,

size = 4.2,

colour = "black",

family = "Times New Roman"

) +

scale_fill_brewer(palette = "Set2") +

labs(

title = "Proportional Coverage of Cattle Egret Research Themes",

subtitle = "Percentage share and sample size (n) for each analytical pillar",

x = "Analytical Pillar",

y = "Percentage of Studies (%)",

caption = "Note: a single study may contribute to multiple analytical pillars."

) +

theme(

text = element_text(family = "Times New Roman", colour = "black", size = 13),

panel.grid = element_blank(),

panel.background = element_rect(fill = "white", colour = NA),

plot.background = element_rect(fill = "white", colour = NA),

axis.line = element_line(colour = "black", linewidth = 0.7),

axis.text.x = element_text(

angle = 0,

hjust = 0.5,

vjust = 1,

colour = "black",

family = "Times New Roman"

),

axis.text.y = element_text(colour = "black"),

axis.title = element_text(colour = "black"),

plot.title = element_text(face = "bold", colour = "black", size = 15),

plot.subtitle = element_text(colour = "black"),

plot.caption = element_text(colour = "black", family = "Times New Roman", size = 11, hjust = 0),

legend.position = "none"

)

ggsave(

filename = "cattle_egret_theme_plot.png",

width = 10,

height = 7,

dpi = 600

)
